# Universal versus conditional day 3 follow-up for children with non-severe unclassified fever at the community level in the Democratic Republic of the Congo: A cluster-randomized, community-based non-inferiority trial

**DOI:** 10.1371/journal.pmed.1002552

**Published:** 2018-04-17

**Authors:** Luke C. Mullany, Elburg W. van Boetzelaer, Julie R. Gutman, Laura C. Steinhardt, Pascal Ngoy, Yolanda Barbera Lainez, Alison Wittcoff, Steven A. Harvey, Lara S. Ho

**Affiliations:** 1 Department of International Health, Johns Hopkins Bloomberg School of Public Health, Baltimore, Maryland, United States of America; 2 International Rescue Committee, Kalemie, Democratic Republic of the Congo; 3 Malaria Branch, Division of Parasitic Diseases and Malaria, Centers for Disease Control and Prevention, Atlanta, Georgia, United States of America; 4 International Rescue Committee, New York, New York, United States of America; London School of Hygiene & Tropical Medicine, UNITED KINGDOM

## Abstract

**Background:**

The World Health Organization’s integrated community case management (iCCM) guidelines recommend that all children presenting with uncomplicated fever and no danger signs return for follow-up on day 3 following the initial consultation on day 1. Such fevers often resolve rapidly, however, and previous studies suggest that expectant home care for uncomplicated fever can be safely recommended. We aimed to determine if a conditional follow-up visit was non-inferior to a universal follow-up visit for these children.

**Methods and findings:**

We conducted a cluster-randomized, community-based non-inferiority trial among children 2–59 months old presenting to community health workers (CHWs) with non-severe unclassified fever in Tanganyika Province, Democratic Republic of the Congo. Clusters (*n =* 28) of CHWs were randomized to advise caregivers to either (1) return for a follow-up visit on day 3 following the initial consultation on day 1, regardless of illness resolution (as per current WHO guidelines; universal follow-up group) or (2) return for a follow-up visit on day 3 only if illness continued (conditional follow-up group). Children in both arms were assessed again at day 8, and classified as a clinical failure if fever (caregiver-reported), malaria, diarrhea, pneumonia, or decline of health status (development of danger signs, hospitalization, or death) was noted (failure definition 1). Alternative failure definitions were examined, whereby caregiver-reported fever was first restricted to caregiver-reported fever of at least 3 days (failure definition 2) and then replaced with fever measured via axillary temperature (failure definition 3). Study participants, providers, and investigators were not masked. Among 4,434 enrolled children, 4,141 (93.4%) met the per-protocol definition of receipt of the arm-specific advice from the CHW and a timely day 8 assessment (universal follow-up group: 2,210; conditional follow-up group: 1,931). Failure was similar (difference: –0.7%) in the conditional follow-up group (*n =* 188, 9.7%) compared to the universal follow-up group (*n =* 230, 10.4%); however, the upper bound of a 1-sided 95% confidence interval around this difference (−∞, 5.1%) exceeded the prespecified non-inferiority margin of 4.0% (non-inferiority *p =* 0.089). When caregiver-reported fever was restricted to fevers lasting ≥3 days, failure in the conditional follow-up group (*n =* 159, 8.2%) was similar to that in the universal follow-up group (*n =* 200, 9.1%) (difference: −0.8%; 95% CI: −∞, 4.1%; *p =* 0.053). If caregiver-reported fever was replaced by axillary temperature measurement in the definition of failure, failure in the conditional follow-up group (*n =* 113, 5.9%) was non-inferior to that in the universal follow-up group (*n =* 160, 7.2%) (difference: −1.4%; 95% CI: −∞, 2.5%; *p =* 0.012). In post hoc analysis, when the definition of failure was limited to malaria, diarrhea, pneumonia, development of danger signs, hospitalization, or death, failure in the conditional follow-up group (*n =* 108, 5.6%) was similar to that in the universal follow-up group (*n =* 147, 6.7%), and within the non-inferiority margin (95% CI: −∞, 2.9%; *p =* 0.017). Limitations include initial underestimation of the proportion of clinical failures as well as substantial variance in cluster-specific failure rates, reducing the precision of our estimates. In addition, heightened security concerns slowed recruitment in the final months of the study.

**Conclusions:**

We found that advising caregivers to return only if children worsened or remained ill on day 3 resulted in similar rates of caregiver-reported fever and other clinical outcomes on day 8, compared to advising all caregivers to return on day 3. Policy-makers could consider revising guidelines for management of uncomplicated fever within the iCCM framework.

**Trial registration:**

ClinicalTrials.gov NCT02595827

## Abstract 2

In a cluster-randomized non-inferiority trial conducted in the Democratic Republic of Congo, Luke C Mullany and colleagues provide evidence of minimal benefit of universal three-day follow-up to children with non-severe unclassified fever.

## Author summary

**Why was this study done?:**

- Many children in low-resource settings presenting with febrile illness to community health workers have no danger signs and are not diagnosed with malaria, pneumonia, or diarrhea, and as a result, are sent home, and caregivers are advised to return on day 3 (after the initial consultation on day 1).
- As many such non-severe unclassified febrile illnesses self-resolve, the advice given to caretakers could be simplified to return only if the illness continues or worsens.
- Updating the guidelines requires evidence that simplified guidelines would not compromise the health status of these children.

**What did the researchers do and find?:**

- We conducted a cluster-randomized, community-based non-inferiority trial in the Democratic Republic of Congo, where children with non-severe unclassified febrile illness received either the current universal return advice or the simplified advice to return conditionally on illness continuation or progression.
- We found evidence that the simplified advice did not result in any increased clinical failure of these children in the first week or first month after first presenting to community health workers.

**What do these findings mean?:**

- Our findings indicate that the health status of children presenting to community health workers with non-severe unclassified fever is unlikely to be compromised if caregivers are advised to return only if the illness continues or worsens.
- An update to the global guidelines for integrated community case management could be considered, as simplified advice regarding return visits may reduce unnecessary follow-up visits, saving resources for families and health workers.

## Introduction

Continued progress in reducing deaths in children under 5 years old in the context of weak health systems depends partially on the extent to which community health workers (CHWs) can rapidly identify and manage sick children in outpatient settings within their community [1–3]. The World Health Organization (WHO) provides guidelines for CHW cadres in low-resource settings to care for sick children aged 2–59 months through implementation of integrated community case management (iCCM) of common childhood illnesses [4,5], an extension of the integrated management of childhood illness (IMCI) approach to the community level. These guidelines prescribe that CHWs assess children through a combination of caregiver-directed questions and examination. For children with malaria, pneumonia, or diarrhea, CHWs provide direct treatment; for children with danger signs (such as inability to drink or breastfeed, vomiting everything, convulsions, or lethargy/loss of consciousness), CHWs refer immediately to a health center. Caregivers of children who present with fever but are neither diagnosed with malaria, pneumonia, or diarrhea nor have danger signs (non-severe unclassified fever) are advised to return with the child for reassessment on day 3 [4,5].

With recent progress in reducing overall childhood mortality and combating malaria burden, and increased availability of new childhood vaccines for pneumonia, strategies for management of febrile children are being reevaluated [6]. This is particularly true for uncomplicated febrile illnesses, which commonly resolve rapidly without any need for treatment, and are thought to largely be attributable to viruses [7–10]. Among 1,000 children presenting with fever at rural and urban outpatient clinics in Tanzania, viral infections dominated (71%), and fevers were nearly universally self-limiting [11]. Evidence of the safety of withholding treatment for malaria rapid diagnostic test (mRDT)–negative febrile children has been demonstrated in a number of studies [1,12,13]. For example, among nearly 1,000 mRDT-negative febrile children in Tanzania randomly assigned to receive or not receive anti-malarials, investigators found only 3 cases of bacteremia, and no serious adverse events or deaths, among untreated children [12]. In Zambia, when mRDT-negative febrile children were provided only an antipyretic, only 8.2% had continuing fever or were reported by their caregiver to be unwell at a follow-up visit 5–7 days later [13].

For febrile children who have no obvious or classified cause of fever and no danger signs on day 1, the current practice recommending universal follow-up on day 3 even in the absence of continued fever or other signs may be unwarranted, potentially adding substantially to CHW and caregiver time and resource costs. The time and effort to undertake unnecessary visits not only increase the work burden on CHWs, who are usually volunteers, but also represent important opportunity costs to caregivers, whose time might otherwise be utilized for important income-generating activities. To ease this burden, a change to iCCM guidelines could be implemented whereby CHWs advise caregivers to return with the child only if signs persist or worsen. Such an update to global iCCM guidelines, however, requires evidence that expectant home-based management does not compromise the health status of these children. We therefore implemented a cluster-randomized, community-based trial to examine the non-inferiority of advising caregivers to return with mRDT-negative febrile children without danger signs only in cases where the illness progresses or signs do not resolve, compared with universal follow-up (independent of sign resolution).

## Methods

### Study area

**Fig 1:**
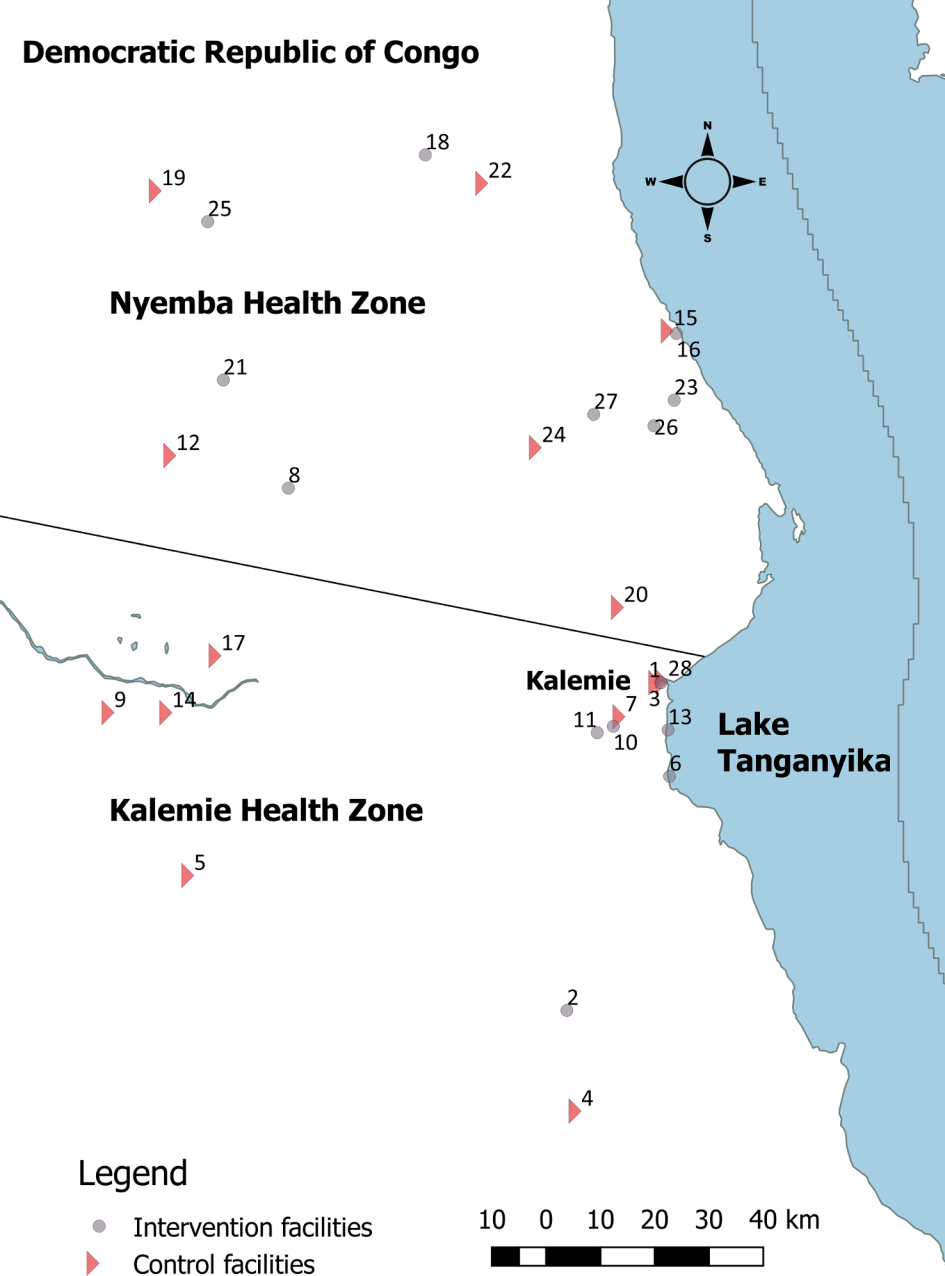
Map of study area. Intervention, conditional follow-up; control, universal follow-up.

We conducted this study in the sparsely populated, southeastern Tanganyika Province of the Democratic Republic of the Congo (DRC), where malaria is endemic [14] and the frequency of formal care-seeking within public sector facilities is low [15]. The International Rescue Committee, in joint collaboration with the Ministry of Public Health, has been implementing the Rapid Access Expansion Program (RAcE) (funded by Global Affairs Canada, administered by WHO) to train and deploy CHWs to deliver iCCM in 11 health zones in Tanganyika Province. CHWs in this setting are not paid a salary, but receive a bicycle from the iCCM program to assist with travel to and from their linked health center. Services provided by CHWs are free of charge to community members. Two RaCE-participating health zones (Kalemie and Nyemba, total CHW catchment area population ~168,000) were selected as the geographic area for our study (Fig 1).

### Study design and aims

Further details of our cluster-randomized non-inferiority trial have been published elsewhere [16]. Briefly, each of the 28 health areas (14 in each zone) consisted of a health center and a team of 1 to 18 associated CHWs: literate, locally resident volunteers who received 5 days of training on the iCCM algorithm and other core competencies (e.g., communication and recording) as per DRC Ministry of Public Health guidelines. We aimed to determine if the risk of clinical deterioration (“failure”) among children aged 2–59 months presenting to these CHWs with non-severe unclassified fever was similar between (1) those advised to follow up with the CHW on day 3 regardless of the child’s condition (universal follow-up) and (2) those advised to follow-up on day 3 only if illness did not resolve (conditional follow-up). Failure was assessed on day 8, and was defined as the child having caregiver-reported fever, 1 of the 3 CHW-treatable illnesses (i.e., malaria, diarrhea, or pneumonia), a referable danger sign, hospitalization, or death. We hypothesized that 5% of children would meet this definition under current guidelines (universal follow-up) and that the true rate of failure among children receiving the updated follow-up advice (conditional follow-up) would be 6%. Our a priori–defined non-inferiority margin for the conditional follow-up approach was 4 percentage points; specifically, conditional follow-up would be considered non-inferior if the upper bound of a 1-sided 95% confidence interval around the absolute difference in outcome rate (conditional follow-up minus universal follow-up) did not exceed 4%.

### Randomization

We used restricted randomization balanced on zone and health area estimates of (1) population size, (2) prior 6-month likelihood of mRDT-negative febrile children (number of children mRDT negative/under-5 population), and (3) geographic distance from CHW to zonal health center to allocate the 28 health areas (and all the CHWs and the children they enrolled within these areas) to either universal or conditional follow-up advice (also see sample size section below) [16].

### Eligibility and intervention

During the enrollment period for this study, any febrile child with neither danger signs nor a CHW-treatable illness (malaria, pneumonia, or diarrhea) was eligible; at initial presentation (day 1) all caregivers were advised to seek follow-up care with the CHW if signs worsened. The CHW then provided the cluster-specific advice about when to return for a follow-up visit. Specifically, CHWs in the universal follow-up group advised all caregivers to come back with the child on day 3, while those in the conditional follow-up group advised a return visit on day 3 only if signs remained the same or worsened.

### Consent, enrollment, and data collection

CHWs verbally requested permission to subsequently (i.e., on day 8) visit the child and caregiver at home, at which point they would explain the study and obtain consent to participate; for those agreeing to this initial recruitment, the CHW notified his/her health area data collector of the eligible child. On the scheduled day 8 visit, the CHW and data collector jointly visited the home, obtained oral informed consent, enrolled the child, and conducted a standard assessment to record outcome and covariate data. The data collector recorded axillary temperature, respiratory rate, and mid-upper arm circumference (MUAC), then the CHW assessed the child using the standard iCCM algorithm. At this visit, any child with reported fever, or who was treated by the CHW for malaria, diarrhea, or pneumonia, or who had a referable danger sign was visited again 2 weeks (and, if necessary, again 4 weeks) later; procedures on repeat visits were identical to those on day 8. Final vital status (alive/died) of all enrolled children was recorded on day 31, regardless of the number of follow-up visits conducted. An additional structured questionnaire was administered (typically on day 8) to elicit information on household demographic and socioeconomic variables, and care-seeking before and after the initial visit to the CHW. All data were collected on paper forms, and identified with a unique 6-digit code, allowing linkage of data for each individual child while maintaining anonymity of child and caregiver identity.

### Quality control

CHWs were fully trained on study procedures, including eligibility determination and delivery of the cluster-appropriate follow-up advice. Each week, supervising data collectors checked the iCCM registers maintained by CHWs to ensure that all eligible children were identified; these measures were supplemented by periodic spot checks of registers by senior field research staff, who also conducted periodic direct observation of data collector field work. Paper forms were checked for accuracy and completeness by a data officer, then entered twice into a secure online database (REDCap) [17] using customized data entry screens with built-in validation checks. Additional details have been previously published [16].

### Sample size

We estimated that the true cluster-specific failure rates would vary between 1.5% and 8.5%, implying a coefficient of variation of approximately 0.35. Based on programmatic data from January to June 2015, we anticipated that of approximately 493 mRDT-negative fever cases per month across the 28 clusters, 70% would be eligible (i.e., after excluding children with danger signs or treatable conditions), leading to an annualized average cluster size of 148 eligible children, of which 90%, or 133, would be enrolled. We estimated that 12 health areas per group would be required to detect non-inferiority (i.e., 1-sided 5% significance) with 80% power [18,19], but elected to include all 28 available health areas (14 per group), thus increasing our a priori–estimated power to 86.8%. Soon after initiating enrollment, our estimate of total per cluster yield was revised to 152 (further increasing our estimated power), and leading to a final anticipated enrollment of 4,270, or 2,135 children per group.

### Analysis

We first assessed randomization balance by comparing the distribution of child, maternal, household, socioeconomic, and CHW characteristics between the two groups, and noted any that appeared unbalanced. We next examined the proportion of children in each group whose caregivers reported receiving follow-up advice congruent with the allocation of their corresponding CHW, and the proportion of day 8 follow-up visits (i.e., outcome assessments) that occurred within ±24 hours of the scheduled date (i.e., between day 7 and 9, inclusive). The subset of children meeting both these criteria defined the group for conducting per-protocol analysis, which was our primary analytic approach, given the non-inferiority design. The primary outcome, failure at day 8, was defined as at least 1 of the following: (1) caregiver reports that the child currently has fever, (2) CHW classification of diarrhea, pneumonia, or malaria, (3) presence of a danger sign, (4) hospitalization, or (5) death. We estimated the difference in the proportion meeting this definition across the study arms (conditional minus universal) along with a 1-sided 95% confidence interval using a binomial regression model with an identity link function, and conducted robust standard error estimation to account for the clustered design.

To increase the specificity of our outcomes, we additionally examined 3 alternate definitions of failure, whereby the first component (caregiver report of fever) in the above composite definition was modified. The progressively specific modifications were as follows: definition 2 required that caregiver-reported fever had been present for ≥3 days, definition 3 used measured axillary temperature ≥ 38.0°C in place of the caregivers’ report (objective clinical failure), and definition 4 eliminated fever from the composite definition of failure. These 3 additional definitions were constructed and analyzed in a manner identical to the first definition. Among all 4 definitions, the first 3 were prespecified analyses. During a post-study analytic workshop in conjunction with discussions with the data and safety monitoring board, the fourth definition (requiring that at least 1 of death, hospitalization, referral for danger signs, or a CHW-treated illness was present) was added, in an effort to further increase specificity given the higher than expected rates of caregiver-reported fever. The estimation of the difference in failure rate between the 2 groups was repeated for each definition, adjusting for (i.e., including as fixed effects) variables appearing imbalanced. An intent-to-treat analysis, whereby the above-mentioned per-protocol requirements were removed, was also conducted. Finally, given the relatively small number of clusters, we also conducted a cluster-level analysis of the primary outcome definitions, using a *t* test to estimate the difference in the mean cluster-level proportions.

Secondary outcome analysis was descriptive; we describe the clinical presentation at enrollment, care-seeking patterns before and after enrollment, and longer-term outcomes 2 and 4 weeks later (at day 15 and day 29) of children that were classified as “failed” at day 8. All analyses were conducted using R (https://cran.r-project.org/) and Stata 14.2 (StataCorp, College Station, TX).

### Ethical approval

The study protocol was approved by the Johns Hopkins Bloomberg School of Public Health’s Institutional Review Board (No. 6608), and the Ethical Committee of the Ministry of Public Health in Kinshasa, DRC (No. 001/CNES/CNES/SR/03/2015). US Centers for Disease Control and Prevention investigators participated under a non-engaged determination from their agency’s Office for Human Research Protections.

## Results

### Participation

**Fig 2:**
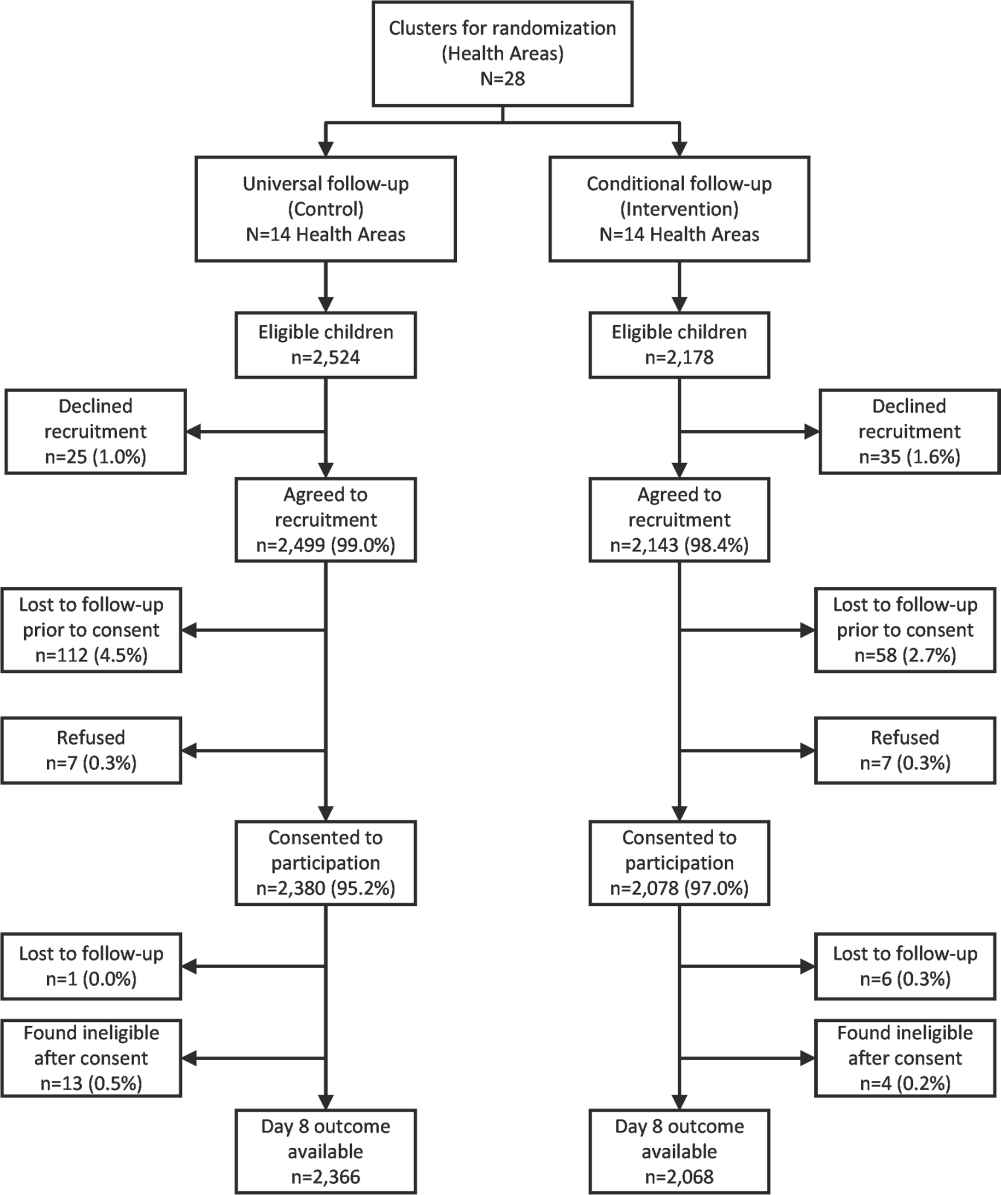
Study participant flowchart.

Between October 12, 2015, and November 28, 2016, 233 CHWs working in Kalemie and Nyemba health zones identified 4,702 eligible children. For approximately 1.3% (*n =* 60) of these children, their caregivers declined to provide preliminary permission; for the remaining 4,642 eligible children, 14 of their caregivers refused to participate in the day 8 follow-up, and 170 (3.7%) were lost to follow-up between initial identification as eligible and the follow-up visit, while the caregivers for the remaining 4,458 children were met and provided consent. Refusal and loss to follow-up rates did not differ between the groups. After consent and upon extraction of information recorded by the CHW at enrollment, 17 children were excluded (13 and 4 in the universal and conditional follow-up groups, respectively) because they were found to meet the age-specific criteria for pneumonia and thus should not have been eligible. In the universal and conditional follow-up arms, respectively, there were 2,366 (mean age 2.1 years) and 2,068 (mean age 2.0 years) children who were assessed at the day 8 visit and provided information on final outcome (Fig 2).

### Randomization balance

The groups were largely balanced on a range of child, maternal, household, and CHW characteristics (Table 1). Maternal literacy was slightly higher in the conditional follow-up group, while the distribution of self-reported religious affiliation, source of household water, and availability of electricity differed only slightly between the groups. CHWs to whom children initially presented were similar across groups in terms of marital and educational status and time since receiving CHW training, but a greater proportion presented to female CHWs in the conditional than in the universal follow-up group (28.3% versus 15.0%). There were no substantive differences between the groups in the clinical and care-seeking descriptors of children at enrollment (Table 2).

**Table 1:** Distribution of child, maternal, household, and CHW characteristics between universal and conditional follow-up groups.

Characteristic	Universal follow-up		Conditional follow-up	
	*N*	Percent	*N*	Percent
**Child characteristics**				
**Total number of children**	2,366	100.0	2,068	100.0
**Age of child**^**1**^				
[2.0 months–1.0 years)	636	26.9	622	30.1
[1.0–2.0 years)	526	22.2	486	23.5
[2.0–3.0 years)	425	18.0	377	18.3
[3.0–4.0 years)	366	15.5	256	12.4
[4.0–5.0 years)	412	17.4	324	15.7
**Sex**^**2**^				
Male	1,172	49.6	1,000	48.4
Female	1,193	50.4	1,065	51.6
**Mother characteristics**				
**Age of mother**^**3**^				
[15–20 years)	161	7.0	191	9.6
[20–25 years)	460	20.0	405	20.1
[25–30 years)	726	31.5	556	27.8
[30–35 years)	563	24.5	415	20.8
[35–40 years)	242	10.5	265	13.3
[40 years and older)	151	6.5	168	8.4
**Marital status**^**4**^				
Married	2,136	90.8	1,868	90.9
Single/divorced/widow	217	9.2	188	9.1
**Number of pregnancies**^**5**^				
1–2	530	22.6	427	20.8
3–4	940	40.2	791	38.6
5–7	713	30.5	621	30.3
8 or more	158	6.7	213	10.4
**Maternal education**^**6**^				
None/minimal	1,446	61.3	1,160	56.3
Primary	600	25.4	619	30.0
Secondary or more	314	13.3	283	13.7
**Maternal literacy**^**7**^				
None	1,525	64.6	1,177	57.1
Reads with difficulty	654	27.7	616	29.9
Able to read	182	7.7	269	13.1
**Household characteristics**				
**Religion**^**8**^				
Christian	1,468	63.3	1,485	72.3
Muslim	124	5.4	121	5.9
Kitabala	407	17.6	198	9.6
Other	319	13.7	250	12.1
**Primary water source**^**9**^				
Piped water (indoor/shared/public)	295	12.5	53	2.6
Drilled well/pump	42	1.8	228	11.1
Protected dug well/spring water	148	6.3	422	20.5
Unprotected dug well/spring water	1,192	50.6	410	19.9
Surface water (river/lake/pond)	591	25.1	887	43.0
Collected rainwater	85	3.6	60	2.9
Other	4	0.2	1	0.0
**Type of toilet**^**10**^				
Flush (sewer/tank/latrine)	56	2.4	8	0.4
Improved pit latrine	44	1.9	61	3.0
Basic latrine	602	25.5	399	19.4
None (open defecation)	1,437	60.9	1,453	70.5
Other	219	9.3	140	6.8
**Electricity available**^**11**^	180	7.6	28	1.4
**Bicycle owned by household**^**12**^	483	20.5	518	25.2
**Characteristics of CHW providing care**				
**CHW sex**^**13**^				
Male	1,986	85.0	1,471	71.7
Female	349	15.0	581	28.3
**CHW marital status**^**14**^				
Married	2,167	93.3	1,906	92.9
Single/divorced/other	156	6.7	146	7.1
**Time since CHW training**^**15**^				
<1 year	432	18.6	359	17.5
≥1 to <2 years	841	36.1	760	37.0
≥2 years	1,054	45.3	933	45.5

Missing *N* (percent) in universal and conditional follow-up groups, respectively

^1^1 (0.0%), 3 (0.2%)

^2^1 (0.0%), 3 (0.2%)

^3^63 (2.7%), 68 (3.3%)

^4^13 (0.5%), 12 (0.5%)

^5^25 (1.0%), 16 (0.%)

^6^6 (0.2%), 6 (0.3%)

^7^5 (0.2%), 6 (0.3%)

^8^48 (2.0%), 14 (0.7%)

^9^8 (0.3%), 7 (0.3%)

^10^8 (0.3%), 7 (0.3%)

^11^11 (0.5%), 9 (0.4%)

^12^11 (0.5%), 9 (0.4%)

^13^31 (1.3%), 16 (0.8%)

^14^43 (1.8%), 16 (0.8%)

^15^39 (1.6%), 16 (0.8%).

CHW, community health worker.

**Table 2:** Clinical and care-seeking descriptors of enrolled children at initial presentation to CHW.

Characteristic	Universal follow-up		Conditional follow-up	
	*N*	Percent	*N*	Percent
**Duration of fever**^**1**^				
0–1 days	598	25.4	379	18.3
2 days	1,157	49.0	1,157	56.0
3 days	404	17.1	398	19.3
4 or more days	201	8.5	131	6.4
**MUAC classification**^**2**^				
Green (>135 mm)	1,962	98.7	1,853	99.5
Yellow (125–135 mm)	25	1.3	9	0.5
**Cough**^**3**^	256	10.8	314	15.2
**Duration of cough**^**4**^				
0–1 days	58	22.7	73	23.3
2 days	88	34.4	118	37.6
3 days	68	26.6	80	25.5
4 or more days	42	16.4	43	13.7
**Respiratory rate ≥ 40 breaths/minute**^**5**^	35	16.8	50	16.8
**Sought care prior to CHW visit**^**6**^	111	4.7	43	2.1

Missing *N* (percent) in universal and conditional follow-up groups, respectively

^1^6 (0.3%), 3 (0.2%)

^2^379 (16.0%), 206 (10.0%)

^3^3 (0.1%), 4 (0.2%)

^4^0 (0.0%), 0 (0.0%) (measured only in those presenting with cough)

^5^47 (18.4%), 16 (5.0%) (measured only in those presenting with cough)

^6^4 (0.2%), 3 (0.2%).

CHW, community health worker; MUAC, mid-upper arm circumference.

In both groups, approximately three-quarters of caregivers indicated that the fever had been present for 2 days or fewer. Caregivers were slightly more likely to report cough along with the presenting fever in the conditional follow-up group (314/2,064; 15.2%) compared with the universal follow-up group (256/2,363; 10.8%). Care-seeking prior to contacting the CHW was rare in both groups, with 4.7% (111/2,362) and 2.1% (43/2,065) reporting seeking some care (predominately from non-qualified providers including family members, neighbors, or traditional healers) prior to initial presentation to the CHW in the universal and conditional follow-up groups, respectively.

### Advice given and return visits

More than 98% of caregivers reported that the CHW gave them follow-up advice that was congruent with the allocation of the cluster: in the universal follow-up group, 2,352 of 2,366 caregivers (99.4%) reported that the CHW indicated they should return on day 3, while in the conditional follow-up group, 2,044 of 2,068 (98.8%) reported being instructed to return on day 3 if the child’s illness had not resolved (Table 3).

**Table 3:** Advice given by CHWs, return visits made, and reasons for returning to the CHWs.

Response	Universal follow-up		Conditional follow-up	
	*N*	Percent	*N*	Percent
**Advice given by CHW (as reported by caregiver)**				
Come back on day 3	2,352	99.4	19	0.9
Come back if still sick on day 3	12	0.5	2,044	98.8
Don’t recall	2	0.1	5	0.2
**Return was made**	1,861	78.7	187	9.0
**Reason for return visit**				
Child’s illness worsened	43	2.3	30	16.0
Child was not improving	31	1.7	115	61.5
CHW directed me to return	1,778	95.5	41	21.9
Other/missing	9	0.5	1	0.5

CHW, community health worker.

As a result, the overall rate of return to the CHW between enrollment and day 8 was substantially higher in the universal follow-up group (1,861/2,366, 78.7%) than the conditional follow-up group (187/2,068, 9.0%). In the conditional follow-up group, of the 187 caregivers who returned with their child, 41 reported returning because the CHW directed them to return (21.9% of all returnees, but only 2.0% of the group overall). Caregivers’ reports of return visits to the CHW matched very closely with the CHWs’ own records; sensitivity and specificity of the caregiver’s report compared to gold standard CHW records were 95.7% and 98.2%, respectively. Less than 3% of caregivers reported that their child received any anti-malarials, antibiotics, or medications for diarrhea during the period following the initial visit to the CHW, and there were no differences between the groups.

### Primary outcome analysis

Among all enrolled children, 93.4% (4,141/4,434) met the per-protocol management definition and were included in the primary outcome analysis. Among those not meeting this definition (*n =* 293), this was because the day 8 visit occurred late (i.e., >9 days, *n =* 237, 80.9%) or early (i.e., <6 days, *n =* 21, 7.2%), or caregivers reported receiving advice incongruent with the allocation (*n =* 29, 9.9%). Among the 4,141 included children, the overall rate of failure using the primary outcome definition was 10.1%; cluster-specific rates varied substantially across the 28 clusters (mean = 11.6%, SD: 8.2), resulting in a coefficient of variation of 0.71. The total number (and proportion) of children meeting the primary outcome definitions was similar regardless of the advice provided (Table 4).

**Table 4:** Proportion of children meeting the failure definitions and individual components of the failure definitions at day 8 visit (per-protocol analysis).

Failure definition or component	Universal follow-up (*n =* 2,210)		Conditional follow-up (*n =* 1,931)		Difference (95% CI)^1^	*p*-Value^2^
	*N*	Percent	*N*	Percent		
**Failure definition**						
1. Death, hospitalization, referral for danger signs, malaria, diarrhea, pneumonia, OR caregiver’s report of fever^3^	230	10.41	188	9.74	−0.67% (−∞, 5.05%)	0.089
2. Death, hospitalization, referral for danger signs, malaria, diarrhea, pneumonia, OR caregiver’s report of fever ≥3 days	200	9.05	159	8.23	−0.82% (−∞, 4.08%)	0.053
3. Death, hospitalization, referral for danger signs, malaria, diarrhea, pneumonia, OR axillary temperature ≥ 38.0°C	160	7.24	113	5.85	−1.39% (−∞, 2.52%)	0.012
4. Death, hospitalization, referral for danger signs, malaria, diarrhea, OR pneumonia	147	6.65	108	5.59	−1.06% (−∞, 2.85%)	0.017
**Individual components of composite failure definitions**						
Fever						
Fever reported by caregiver	200	9.05	151	7.82		
Fever reported by caregiver, fever ≥3 days	116	5.25	97	5.02		
Axillary temperature ≥ 38.0°C	31	1.40	21	1.09		
CHW-treatable conditions						
Malaria	105	4.75	78	4.04		
Pneumonia	14	0.63	15	0.78		
Diarrhea	30	1.36	4	0.21		
Danger signs	13	0.59	5	0.26		
Hospitalization	9	0.41	11	0.57		
Death	2	0.09	2	0.10		

^1^One-sided 95% confidence interval, accounting for clustered design of study.

^2^*p*-Value for test of non-inferiority.

^3^Primary outcome definition.

CHW, community health worker.

For our first definition (primary), the rate of failure in the conditional follow-up group (9.74%, 188/1,931) was 0.67% lower than in the universal follow-up group (10.41%, 230/2,210), but the upper bound of the 1-sided 95% confidence interval was 5.1%, slightly above our non-inferiority margin (*p =* 0.089). Failure rates were similar between the 2 groups for all alternative definitions. When caregiver-reported fever was restricted to fevers lasting ≥3 days, the upper bound of the 1-sided 95% confidence interval (4.1%) exceeded the non-inferiority margin; when utilizing axillary temperature to classify fever, the upper bound was 2.2%. When clinical failure was defined in terms of the health outcomes death, hospitalization, referral for danger signs, or a CHW-classified and -treated condition (malaria, pneumonia, or diarrhea), the absolute failure proportions were 5.59% and 6.65% in the conditional and universal follow-up groups, respectively (absolute difference: −1.06%; 95% CI: −∞, 2.85%; *p =* 0.017).

The likelihood of individual components of the failure definition was likewise similar in both groups (Table 4). Overall, caregivers reported the child had fever at the time of follow-up in 200 (9.1%) and 151 (7.8%) cases in the universal and conditional follow-up groups, respectively. The overall proportion of children with fever reduced to about 5.1% overall considering only children whose caregiver-reported fever had been continuing for ≥3 days, and reduced further to 1.3% when fever was defined as axillary temperature ≥ 38.0°C (measured by the data collectors). In all, 105 (4.8%) and 78 (4.0%) children in the universal and conditional follow-up groups, respectively, were classified with malaria; other findings were rarely seen. There were 20 hospitalizations (9 and 11 in the universal and conditional follow-up groups, respectively) and 4 deaths (2 and 2, respectively) within 8 days.

Estimates of the absolute difference in failure rates between the groups, and the corresponding upper bounds of 1-sided 95% confidence intervals, did not change in any substantive way when conducting an intent-to-treat analysis (S1 Table). Similar results were obtained when adjusting for unbalanced variables or estimating outcomes using a cluster-level analysis (S2 Table).

### Follow-up through 2 and 4 weeks

Of the 418 children who met the primary definition of failure at the day 8 visit, 385 (92.1%) had a follow-up visit at 2 weeks (day 15). Of these, 13.8% met the same definition of failure at day 15 (30/217) in the universal follow-up group, compared with 10.7% (18/168) in the conditional follow-up group. Among these 48 failures at 2 weeks (day 15), 45 visits were completed at 4 weeks, with 2 of 28 (7.1%) in the universal follow-up group and 1 of 17 (5.9%) in the conditional follow-up group meeting the failure definition. When considering all children in the per-protocol analysis over the entire follow-up period (including day 8, 2 weeks, 4 weeks, and day 31 [for vital status] follow-up), there were 151/2,210 (6.83%) and 111/1,931 (5.75%) instances of the most specific components of failure (death, hospitalization, referral for danger signs, or classification with malaria, pneumonia, or diarrhea) in the universal and conditional follow-up groups, respectively (absolute difference: −1.13%; 95% CI: −∞, 2.89%; *p =* 0.018).

## Discussion

In these rural communities of southeastern DRC, caregivers depend heavily on government-supported CHWs to respond to childhood illnesses, most commonly febrile illnesses. Caregivers and CHWs alike are best served if frameworks such as iCCM containing guidelines for fever management are based on the best available evidence, and promote efficiency in use of scarce resources, including CHW and caregiver time. In this study, we found that advising caregivers of children under 5 years old presenting with uncomplicated fever to return on day 3 only if signs continued or worsened resulted in similar rates of clinical failure in the week after presentation when compared with current guidance of universal follow-up visits on day 3. Compared to caregiver-reported fever, the statistical strength of the evidence for non-inferiority was greater for measured fever, danger signs requiring referral, or other clinical outcomes (CHW-treatable diagnoses, hospitalization, or death). We also observed consistency of parameter estimates (i.e., little difference in failure rates) across all definitions, when considering individual components of the clinical failure definitions, and when taking an intent-to-treat versus per-protocol analytic approach. This study was planned in conjunction with a similar sister study in Southern Nations, Nationalities, and Peoples’ Region, Ethiopia (ClinicalTrials.gov identifier NCT02926625), where the prevalence of malaria is much lower. The results were similar, with an absolute difference of –3.81% (95% CI: −∞, 0.65%) between the conditional and universal follow-up groups for the primary outcome [20], indicating that the conditional approach was minimally inferior or non-inferior to universal follow-up across at least 2 different settings of malaria epidemiology and health systems.

Approximately 90% of illnesses resolved within a week, in line with findings from some other studies of mRDT-negative fevers where artemisinin-based combination therapies were withheld [1,13], though this proportion is lower than the 98%–99% resolution of other reports [3,21]. In our study, on day 8, 8.5% of caregivers overall reported that the child had fever; however, only 5.1% reported fever that had been present for at least 3 days, suggesting that some of those with fever on day 8 might have had a new illness unrelated to the initial presentation. More than half (52.1%) of all failures and almost three-quarters (71.6%) of objective clinical failures were due to malaria, a slightly higher rate than in other studies [1,21], likely reflecting higher levels of transmission at this site.

In some communities, even when current guidelines are followed and CHWs advise universal follow-up on day 3, the actual return visit rate may be substantially lower than 100%. Prior to conducting the trial, we posited that adherence to the universal follow-up advice might be poor. The return visit rate for children in this group, however, approached 80%, but this is not necessarily generalizable to other settings. In our setting, some CHWs in the universal follow-up group reported actively seeking out children for their follow-up visit; further investigation would be needed to understand what the return rate would be in settings where CHWs did not actively follow up children on day 3. The rate of return does not, however, directly affect the interpretation of the original question, which is focused on the actions/guidance of the CHW and does not depend on the response of the caregivers. This can be understood by considering the extreme case: if all caregivers returned only if the child continued to be sick, regardless of universal or conditional follow-up advice, then the natural conclusion would be that the advice given is irrelevant (i.e., equivalent) relative to health status at day 8.

Along these lines, CHWs in some countries typically refer febrile children with a negative mRDT to health facilities. However, caregivers in many cases do not complete the referral [22], perhaps perceiving their child’s illness to be insufficiently serious to comply; this might undermine CHW referral advice when truly warranted (e.g., children with danger signs) [23]. Simplifying guidelines about CHW advice about management of non-malarial febrile illness could reduce confusion and increase compliance with CHW referral advice for severe cases. Additionally, reducing unnecessary follow-up visits and referrals may save resources, both for families and for CHWs and facility health workers. We note that this conclusion applies only to the subset of febrile children presenting to CHWs who are not treated or referred for danger signs; when this subset is a very small proportion of those visiting CHWs, the savings in terms of time and resources would be less.

There were a number of limitations to this study. We underestimated the overall rate of clinical failure when including caregiver’s report of fever in the definition; at the design stage we anticipated that approximately 5% of children would be classified as failures at the day 8 follow-up, yet the actual rate was closer to double this. As a result, the non-inferiority margin, which was set a priori at 4%, ultimately represented a smaller relative increase in failure than we originally considered as representing non-inferiority. This issue may have arisen in part from the nature of the primary outcome definition; it is possible that caregivers may have overreported fever at 8 days if they perceived a greater likelihood of receipt of medicine, advice, or other material assistance if fever was reported in the presence of the data collector. In general, for all iterations of the clinical failure definition, the substantial variance in cluster-specific rates reduced the precision of our estimates around the difference in rates. Despite this decrease in power, only for the definitions that included caregiver-reported fever did this result in the upper bound of the confidence interval exceeding the non-inferiority margin. Finally, this region of DRC remains continuously affected by ongoing security concerns due to low-level conflict between multiple armed groups. While we were largely successful in conducting the study throughout the planned data collection period, slowly increasing levels of tensions in some areas led to restricted movement for caregivers, CHWs, and data collectors, leading to slightly lower recruitment rates as the study progressed.

### Conclusion

A driving concern underlying the choice between universal and conditional follow-up recommendations for children with uncomplicated fever is that under the less intense conditional follow-up approach, a greater proportion of children might suffer clinical outcomes. In the current study, we found largely similar likelihoods of clinical failure regardless of the approach used for follow-up advice, using a series of increasingly restrictive definitions. Specifically, when clinical failure comprised objective outcomes (hospitalization or death) and CHW-observed measures of clinical deterioration (i.e., progression to malaria, pneumonia, or diarrhea, or referable danger signs), our data, in conjunction with the data from Ethiopia, provide evidence that conditional follow-up is non-inferior to universal follow-up. iCCM guidelines could be updated to allow either approach to management of uncomplicated fever, so that decision-making for policy-makers can be responsive to context-specific considerations while still adhering to global recommendations.

## Supporting information

S1 CONSORT Checklist (PDF)

S1 Table Proportion of children meeting failure definitions and individual definition components at day 8 visit (intent-to-treat analysis). (DOCX)

S2 Table Comparison of unadjusted, adjusted, and cluster-level analysis for failure definitions. (DOCX)

S1 Protocol Study protocol. (PDF)

## Abbreviations

CHW: community health worker
DRC: Democratic Republic of the Congo
iCCM: integrated community case management
mRDT: malaria rapid diagnostic test
MUAC: mid-upper arm circumference
RAcE: Rapid Access Expansion Program
WHO: World Health Organization
