# Supplementary material for: Universal versus conditional day 3 follow-up for children with non-severe unclassified fever at the community level in the Democratic Republic of the Congo: A cluster-randomized, community-based non-inferiority trial
Source: PLoS Med. 2018 Apr 17;15(4):e1002552. doi: 10.1371/journal.pmed.1002552 (PMC5903590; doi:10.1371/journal.pmed.1002552)
Supplement: S1 Table — (DOCX) [file pmed.1002552.s002.docx]

**S1 Table: Proportion of children meeting “failure” definition(s) and individual definition elements at Day 8 visit (intent-to-treat analysis)**

|  | **Universal (n=2,366)** | | **Conditional (n=2,068)** | |  |  |  |
| --- | --- | --- | --- | --- | --- | --- | --- |
| **Failure Definitions** | **N** | **%** | **N** | **%** | **Difference** | **95% CI*** | **p-value**** |
| 1. Death, hospitalization, referral for danger signs, malaria, fever, pneumonia, OR mother’s report of fever | 245 | 10.36 | 213 | 10.30 | -0.01% | (-∞, 5.58%) | 0.118 |
| 1. Death, hospitalization, referral for danger signs, malaria, fever, pneumonia, OR mother’s report of fever > 3 days | 214 | 9.04 | 179 | 8.66 | -0.39% | (-∞, 4.42%) | 0.066 |
| 1. Death, hospitalization, referral for danger signs, malaria, fever, pneumonia, OR axillary temperature > 38.0°C | 174 | 7.35 | 127 | 6.14 | -1.21% | (-∞, 2.63%) | 0.013 |
| 1. Death, hospitalization, referral for danger signs, malaria, fever, OR pneumonia | 160 | 6.76 | 120 | 5.80 | -0.96% | (-∞, 2.91%) | 0.017 |
| **Individual Elements of Composite Failure Definitions** |  |  |  |  |  |  |  |
| Fever |  |  |  |  |  |  |  |
| - Fever reported by mother | 215 | 9.09 | 172 | 8.32 |  |  |  |
| - Fever reported by mother, fever > 3 days | 123 | 5.20 | 110 | 5.32 |  |  |  |
| - Axillary temperature > 38.0°C | 35 | 1.48 | 24 | 1.16 |  |  |  |
| CHW Diagnosed/Treatable Conditions |  |  |  |  |  |  |  |
| - Malaria | 116 | 4.90 | 86 | 4.16 |  |  |  |
| - Pneumonia | 15 | 0.59 | 17 | 0.82 |  |  |  |
| - Diarrhea | 31 | 1.31 | 7 | 0.34 |  |  |  |
| Danger Signs | 14 | 0.59 | 6 | 0.29 |  |  |  |
| Hospitalization | 9 | 0.38 | 12 | 0.58 |  |  |  |
| Death | 2 | 0.08 | 2 | 0.10 |  |  |  |

*one-sided 95% confidence interval, accounting for clustered design of study; ** p-value for test of non-inferiority
