# Supplementary material for: Universal versus conditional day 3 follow-up for children with non-severe unclassified fever at the community level in the Democratic Republic of the Congo: A cluster-randomized, community-based non-inferiority trial
Source: PLoS Med. 2018 Apr 17;15(4):e1002552. doi: 10.1371/journal.pmed.1002552 (PMC5903590; doi:10.1371/journal.pmed.1002552)
Supplement: S2 Table — (DOCX) [file pmed.1002552.s003.docx]

**S2 Table: Comparison of unadjusted, adjusted, and cluster-level analysis for failure definitions**

|  | **Unadjusted** | | **Adjusted*** | | **Cluster-level** | |
| --- | --- | --- | --- | --- | --- | --- |
| **Failure Definitions** | **Difference (%_Cond_ - %_Univ_)** | **95% CI** | **Difference (%_Cond_ - %_Univ_)** | **95% CI** | **Difference (%_Cond_ - %_Univ_)** | **95% CI** |
| 1. Death, hospitalization, referral for danger signs, malaria, diarrhea, pneumonia, OR mother’s report of fever | -0.67% | (-∞, 5.05%) | 0.74% | (-∞, 5.77%) | 0.71% | (-∞, 6.11%) |
| 1. Death, hospitalization, referral for danger signs, malaria, diarrhea, pneumonia, OR mother’s report of fever > 3 days | -0.82% | (-∞, 4.08%) | 0.28% | (-∞, 4.51%) | 0.28% | (-∞, 5.09%) |
| 1. Death, hospitalization, referral for danger signs, malaria, diarrhea, pneumonia, OR axillary temperature > 38.0°C | -1.39% | (-∞, 2.52%) | -0.41% | (-∞, 2.84%) | -1.14% | (-∞, 2.73%) |
| 1. Death, hospitalization, referral for danger signs, malaria, fever, OR pneumonia | -1.06% | (-∞, 2.85%) | -0.44% | (-∞, 3.00%) | -0.78% | (-∞, 3.00%) |

* Adjusted model included sex of CHW providing initial and follow up care, religion, electricity, water source, and maternal literacy as fixed effects in the binomial regression model.
