# Supplementary material for: Universal versus conditional day 3 follow-up for children with non-severe unclassified fever at the community level in the Democratic Republic of the Congo: A cluster-randomized, community-based non-inferiority trial
Source: PLoS Med. 2018 Apr 17;15(4):e1002552. doi: 10.1371/journal.pmed.1002552 (PMC5903590; doi:10.1371/journal.pmed.1002552)
Supplement: S1 Protocol — (PDF) [file pmed.1002552.s004.pdf]

Universal vs. Conditional 3-day follow up visit for children with unclassified fever at the community level: a cluster-randomized, community-based non-inferiority trial in Kalemie, Democratic Republic of Congo

Final Study Documentation

SECTION I: Introduction

The TRAction project (Transforming Research in Action) that is funded by USAID through URC, aims to provide the scientific evidence to improve and strengthen maternal and child health programs. Currently TRAction is funding two studies in the DRC and Ethiopia to determine if a follow-up visit to a Community Health Worker (CHW) is necessary for children who have a simple fever. In the DRC, the study will be implemented by International Rescue Committee (IRC) in partnership with Johns Hopkins Bloomberg School of Public Health (JHSPH) in Tanganyika District, Katanga Province.

Currently, the WHO protocol recommends systematic monitoring for children with a simple fever. Every child that visits a CHW with a simple fever, is advised to return to the CHW after 2 days, even if the child has been cured of fever and is now healthy. The systematic follow up visit can waste time and resources of the CHW as well as of the family of the child. To explore whether this systematic follow-up visit on Day 3 (Day 1 is the day when the child first came to the CHW for care) is necessary, this study compares a systematic follow-up visit with a conditional follow up visit. Regarding the conditional follow-up visit, the CHW advises the caregiver to return after three days to the site only if the child is still febrile or if the child has developed other symptoms or danger signs. This study aims to evaluate the non-inferiority of the systematic follow-up visit that is recommended by WHO compared to a conditional follow-up visit for children with a simple fever. In addition, this study aims to describe the health status of children with a simple fever over the course of 30 days; to determine the proportion of children who return to the CHW for a follow-up visit on day 3; and to determine the proportion of children who develop danger signs and who are referred to a health center for care.

The eligibility of children for enrollment in this study is determined during the first visit of the child to the CHW. The eligibility criteria include:

• The child is aged 2-59 months

• The child has fever

• The child does not have malaria (RDT-)

• The child does not have pneumonia

• The child does not have diarrhea

• The child is not have any danger sign

• The child is referred not referred to a health center for care

The study will be conducted in two health zones, namely Kalemie and Nyemba. In each of the health zones, 14 health areas were selected to be included in the study. All 28 health areas will be randomly assigned to two arms of the study, each study arms containing 14 health areas. In one study arm (consisting of 14 health areas) a systematic follow-up visit will be implemented for children with a simple fever (all caregivers who come to the CHW with a child with a simple fever will be advised to return to the CHW after 3 days). In the other study arm, consisting of 14 different health areas a conditional follow-up visit will be implemented for children with a simple fever (all caregivers who come

to the CHW with a child with a simple fever will be advised to only return to the CHW if the child is still febrile, or if the child develops any danger signs or other symptoms. If the health status of the child improves, the caregivers are advised not to return to the CHW.

The study will be implemented in the health zones of Kalemie and Nyemba from September 2015 until August 2016. During this year, the target is to enroll 4,000 children in the study. Data collection will be conducted by 24 trained data collectors. More than 250 CHWs will be trained on the eligibility criteria of the study, so that they will be able to inform the data collector when an eligible child presents at the CHW.

To monitor the health of children in both arms of the study, the children will be followed for 30 days. Enrolled children will be visited at their home by a CHW and a data collector 6 days after their first visit to the CHW during which the eligibility of the child was determined (a house visit on Day 7). If the child is still febrile and/or referred for care to a health center during this Day 7 visit, the child will be visited on Day 14 by the CHW and the data collector. If the child is still febrile and/or referred for care to a health center during the Day 14 visit, the child will be visited again on Day 28. During these house visits, the child’s caregiver will be asked questions related to the health of the child, and the child will be examined by the CHW.

In addition to the quantitative phase of the project, we would also like to better understand how CHWs and caregivers perceive the universal and the conditional follow-up visit. Qualitative research methods will allow JHSPH and IRC to obtain in-depth and rich information regarding the perceptions of CHWs and caregivers, in order to learn more about their experiences with the universal and the conditional follow-up visits for children with unclassified fever. This part of the study was implemented in 16 health areas in the health zones of Kalemie and Nyemba. A 3-day training of data collectors was organized, and the subsequent data collection was conducted during one month.

After one year of data collection, researchers from the IRC and JHSPH will analyze the collected data regarding the health status of the enrolled children, and the non-inferiority of a systematic follow-up visit for children with a simple fever will be evaluated. Given the limited evidence currently exists, the objective of this study is to assess which of the two procedures (systematic versus conditional follow up visit) is the most beneficial for children's health. The study that will be conducted by the IRC and JHSPH in Tanganyika District, Katanga Province, will strengthen the implementation of community management of cases of simple fever in DRC and around the world.

SECTION II - Field Manual for Data Collectors (Main Study)

Purpose of this guide

This document should be used as a guide to refer to when you are in the field conducting data collection activities. In this guide every data collection form that will be used for the TRAction project is explained. For each form, all the questions are explained and guidance is provided on how to fill out the forms.

Data Collection Forms TRAction

Form 01	Eligibility Tracking Log
Form 02	SMS and Scheduling Log
Form 03	Initial Visit Extraction Form
Form 04	Return Visit Extraction Form
Form 05	Consent Form
Form 06	Day 7 Assessment Form
Form 07	Care-seeking and Demographic Form
Form 08	Extra Home Visit Form

Form 01 – Eligibility Tracking Log

This form enables the data collector and the CHW to keep track of the children that are eligible for this study. This form will be filled out by the CHW.

The Eligibility Tracking Log has 8 lines which enables us to keep track of 8 children that are eligible for this study. For every child that is eligible for enrollment in this study, the CHW will fill out one line of the Eligibility Tracking Log. The Eligibility Tracking Log is ONLY filled out if a child is eligible for this study. If the child is not eligible for this study, the form will not be filled out by the CHW.

When the data collector receives a message from the CHW that there is a child that is eligible, the data collector visits the house of the CHW. During this visit, the data collector verifies the eligibility of the child, and plans the house visit of the eligible child on day 7. Furthermore, the data collector asks the CHW to see Form 01 – Eligibility Tracking Log, the CHW register, and the iCCM Child Form.

On the Eligibility Tracking Log, the CHW fills out the date of the first visit of the child to the CHW (during this visit the CHW determines the eligibility of the child for participation in this study). In addition, the CHW fills out the name of the child, the name of the caregiver, and the village/town of the house where the child lives. On the Eligibility Tracking Log there is a space to fill out the child's identification code (child id). This is the only element of this form that will be filled out by the data collector. All the other elements will be filled out by the CHW.

An important element of Form 01 – Eligibility Tracking Log is the recruitment statement. On the bottom of the form, you will find a short paragraph, the so-called recruitment statement. The CHW needs to read this paragraph out loud to the caregiver in order to ask him/her for permission to visit the child at his/her home on Day 7. This visit will be conducted by the CHW and the data collector. On the far right of every line on the Eligibility Tracking Log you can find a box in which should be noted whether the caregiver gave his/her permission for the house visit on Day 7. If the caregiver refuses the house visit, please note « 0 » in the box. If the caregiver gives permission to conduct the house visit, please note « 1 » in the box.

Elements of Form 01 – Eligibility Tracking Log

Elements :

1. The date of the first visit of the child to the CHW. During this visit the CHW has determined the eligibility of the child to participate in this study.
2. The name of the child
3. The name of the caregiver
4. The village/town of the child's house
5. The ID code of the child (filled out by the data collector)
6. Recruitment statement

Procedure to fill out Form 01 – Eligibility Tracking Log

1. The CHW notes **the date** of the first visit of the child to the CHW. The date can be found in the CHW register. During this first visit, the CHW has determined the eligibility of the child for this study. The CHW uses the boxes on Form 01 to fill out the date, one digit per box. He fills out the

day in the two boxes on the left, the month in the two boxes in the middle, and the year in the two boxes on the right. He uses two digits to note down the day, two digits to note down the month, and two digits to note down the year. For example : 3 July 2016 should be noted on Form 01 as : 03-07-16.

2. The CHW notes the **name of the child** (only if the child is eligible for this study) on the Eligibility Tracking Log so that the child can be identified and followed over the course of 30 days.
3. The CHW notes the **name of the caregiver of the child** (only if the child is eligible for this study) on the Eligibility Tracking Log so that the child can be identified and followed over the course of 30 days.
4. The CHW notes the **name of the village/town** where the child lives on the Eligibility Tracking Log so that the child can be identified and followed over the course of 30 days.
5. The CHW does not fill out the child ID code. When the data collector visits the house of the CHW he fills out the child ID code (only if the child is eligible for this study).
6. The CHW reads the **recruitment statement** out loud for the caregiver, and makes sure the recruitment statement is well understood.
7. If the caregiver refuses a house visit by the CHW and the data collector after 6 days (on Day 7), the CHW notes « 1 » in the box for « **recruitment statement** ». If the caregiver gives permission for a house visit by the CHW and the data collector after 6 days (on Day 7), the CHW notes « 1 » in the box for « **recruitment statement** ».
8. The CHW sends a **text message** to the data collector to inform him that he has identified an eligible child for this study.

Child ID Code

The child ID code is filled out by the data collector. The child ID code contains 6 digits, and is a unique code for every child that is eligible for this study. The data collectors uses the boxes to fill out the code, one digit per box. He fills out the code for the 'Health Area' in the two boxes on the left, the code for the CHW site in the two boxes in the middle, and the code for the child in the two boxes on the right. The data collector uses two digits for the 'Health Area', two digits for the CHW site, and two digits for the child code (the sequence of the child code is based on the 'Health Area'.)'.

For example, if the data collector that works in the 'Health Area' of Fatuma (code : 03), and the CHW site is Auxelia (code : 10) has identified an eligible child. The child that has been identified is the fifth eligible child of the Health Area of Fatuma (05). Therefore, the child ID code is : 03 10 05.

Form 02 – SMS and Scheduling Log

Form 02 – SMS and Scheduling Log is filled out by the data collector when he receives a text message from the CHW. The CHW sends a text message to the data collector when he identifies a child that is eligible for this study. In the text message, the CHW writes the date of the first visit of the child (the visit during which the CHW determines the eligibility of the child for this study), the name of the child, and the name of the CHW site, and the response of the caregiver to the recruitment statement.

The objective of Form 02 – SMS and Scheduling Log is to enable the follow up of eligible children, and to plan the house visit of the child that is enrolled in this study on Day 7, and, if required, on Day 14 and Day 28. In addition, the data collector Records down the vital status of the child on Form 02 (which will be determined on Day 30).

The SMS and Scheduling Log has 6 lines. The form will be used to plan the house visits of 6 children that are eligible for this study. For every eligible child, the data collector fills out a separate line on the form. The SMS and Scheduling Log will only be filled out if a child is eligible for this study.

Elements of Form 02 – SMS and Scheduling Log

Elements:

1. Code of the Health Center
2. Form sequence
3. Date and time when the text message was logged by the data collector
4. Site code and the date that the CHW sent the text message to the data collector
5. Date of the first visit of the child to the CHW (date when the CHW determined the eligibility of the child for this study)
6. Case ID of the child
7. Date planned for the house visit of the child on Day 7 (for all eligible children)
8. Date planned for the house visit of the child on Day 14 (only for children that are still sick and/or are referred for care during the house visit on Day 7)
9. Date planned for the house visit of the child on Day 28 (only for children that are still sick and/or are referred for care during the house visit on Day 14)
10. Date planned for the vital status check of the child on Day 30
11. The vital status of the child on Day 30

Procedure to fill out Form 02 – SMS and Scheduling Log

When the data collector has received a text message from the CHW regarding the eligibility of a child :

1. Record : **the date** when the text message is received. Use the two boxes on the left to Record the day. Write the month in the two boxes in the middle, and Record the year in the two boxes on the right. Use two digits to Record the day, two digits to Record the month, and two digits to Record the year. For example: write 3 July 2016 as : 03 07 16.
2. Record : the **time** when the text message is received. Use the two boxes on the left for the hour, and the two boxes on the right for the minutes. Use two digits to Record the hour, and two digits to Record the minutes. For example : write 23:15.
3. Record : the **Site Code** in the two boxes. Use two digits to Record the Site Code. For example

when one Record the Site of Auxelia, write : 05.

4. Record : the date that is written in the text message by the CHW : « EL Date ». “El Date” means the “date of eligibility” and reflects the date when the CHW determined the eligibility of the child (during the first visit of the child to the CHW, we refer to this visit as “Day 1”). Use the two boxes on the left to Record the day. Write the month in the two boxes in the middle, and Record the year in the two boxes on the right. Use two digits to Record the day, two digits to Record the month, and two digits to Record the year. For example: write 3 July 2016 as : 03 07 16.
5. Record : the **Case ID of the Child**. Please find guidance how to fill out the Case ID for the child below.
6. Record : the **date planned for the house visit of the child on Day 7**. All children enrolled in this study are visited at their house on Day 7. To plan the house visit on Day 7, count 7 days from the day when the CHW determined the eligibility of the child (the date of the first visit of the child to the CHW; this date is written in the text message that the CHW sends to the data collector). If the date of the house visit on Day 7 falls on a Sunday: plan the visit a day earlier, on Saturday. Write the date planned for the house visit of the child on Day 7 in the boxes. Use the two boxes on the left to Record the day. Write the month in the two boxes in the middle, and Record the year in the two boxes on the right. Use two digits to Record the day, two digits to Record the month, and two digits to Record the year. For example: write 3 July 2016 as : 03 07 16.
7. Record: the **date planned for the house visit of the child on Day 14.** If the child still has a fever during the house visit on Day 7, if the child is treated during the house visit on Day 7, or if the child is referred for care to a health center during the house visit on Day 7 : Plan a house visit of the child on Day 14. To plan the house visit on Day 14, count 14 days from the day when the CHW determined the eligibility of the child (the date of the first visit of the child to the CHW; this date is written in the text message that the CHW sends to the data collector). If the date of the house visit on Day 14 falls on a Sunday: plan the visit a day earlier, on Saturday. Write the date planned for the house visit of the child on Day 14 in the boxes. Use the two boxes on the left to Record the day. Write the month in the two boxes in the middle, and Record the year in the two boxes on the right. Use two digits to Record the day, two digits to Record the month, and two digits to Record the year. For example: write 3 July 2016 as : 03 07 16.
8. If the child does not have fever during the house visit on Day 7, if the child is not treated by the CHW during the house visit on Day 7, and if the child is not referred for care to a health center: do not plan a house visit of the child on Day 14 or on Day 28. Instead, contact the CHW on Day 30 to verify the vital status of the child. Write the vital status of the child on Day 30 on Form 02 – SMS and Scheduling Log.
9. Record: the **date planned for the house visit of the child on Day 28**. If the child still has a fever during the house visit on Day 14, if the child is treated during the house visit on Day 14, or if the child is referred for care to a health center during the house visit on Day 14 : Plan a house visit of the child on Day 28. To plan the house visit on Day 28, count 28 days from the day when the CHW determined the eligibility of the child (the date of the first visit of the child to the CHW; this date is written in the text message that the CHW sends to the data collector). If the date of the house visit on Day 28 falls on a Sunday: plan the visit a day earlier, on Saturday. Write the date planned for the house visit of the child on Day 28 in the boxes. Use the two boxes on the

left to Record the day. Write the month in the two boxes in the middle, and Record the year in the two boxes on the right. Use two digits to Record the day, two digits to Record the month, and two digits to Record the year. For example: write 3 July 2016 as : 03 07 16.

10. If the child does not have fever during the house visit on Day 14, if the child is not treated by the CHW during the house visit on Day 14, and if the child is not referred for care to a health center: do not plan a house visit of the child on Day 28. Instead, contact the CHW on Day 30 to verify the vital status of the child. Write the vital status of the child on Day 30 on Form 02 – SMS and Scheduling Log.
11. Record : **date planned for the vital status check of the child on Day 30**. To plan this verification, count 30 days from the day when the CHW determined the eligibility of the child (the date of the first visit of the child to the CHW; this date is written in the text message that the CHW sends to the data collector). Write the date planned for the vital status check of the child on Day 30 in the boxes. Use the two boxes on the left to Record the day. Write the month in the two boxes in the middle, and Record the year in the two boxes on the right. Use two digits to Record the day, two digits to Record the month, and two digits to Record the year. For example: write 3 July 2016 as : 03 07 16.
12. Record : the vital status of the child. The data collector contact the CHW on Day 30 to verify the vital status of the child. If the child is dead, Record « 0 ». If the child is alive, Record « 1 ».

Case ID of the Child

The child ID code is filled out by the data collector. The child ID code contains 6 digits, and is a unique code for every child that is eligible for this study. The data collector uses the boxes to fill out the code, one digit per box. He fills out the code for the 'Health Area' '' in the two boxes on the left, the code for the CHW site in the two boxes in the middle, and the code for the child in the two boxes on the right. The data collector uses two digits for the 'Health Area', two digits for the CHW site, and two digits for the child code (the sequence of the child code is based on the 'Health Area'.

For example, if the data collector that works in the 'Health Area' of Fatuma (code : 03), and the CHW site is Auxelia (code : 10) has identified an eligible child. The child that has been identified is the fifth eligible child of the Health Area of Fatuma (05). Therefore, the child ID code is : 03 10 05.

Form 03 – Initial Visit Extraction Form

Form 03 – Initial Visit Extraction Form is filled out for every child that is eligible for this study (the child has a simple fever – a negative RDT, without diarrhea, without pneumonia, without any danger signs, the child is not referred for care to a health center). This form is used to extract information about the child from the individual iCCM Child Form and CHW register. The CHW fills out an individual iCCM Child Form and his register during the first visit of the child to the CHW. Information that can be found on the individual iCCM Child Form and in the CHW register includes the results of the CHW examination, more specifically the complaints of the child, different measures (such as the MUAC, and the respiratory rate of the child), the result of the RDT (if a RDT was done), information about danger signs and warning signs, the treatment of the child by the CHW, and whether the caregiver and the child have returned to the CHW for a follow-up visit after 2 days (on Day 3).

Form 03 – Initial Visit Extraction Form is filled out by the data collector at the house of the CHW on the same day as the house visit of the child on Day 7 takes place.

Elements of Form 03 – Initial Visit Extraction Form

Header : The following elements are included in the header of the form : the date when the form is filled out by the data collector, the data collector's ID code, the code of the health center, the case ID for the child, the name of the child, the name of the caregiver. The data collector also Records whether the child has received a case ID before (if the child has been enrolled in this study before), If the child has received a case ID previously, the data collector Records this code on form 03 in the boxes.

Sections : This form has 2 sections, each section is explained below. The sections of this form are:

- Section A : Information from CHW register on the day of recruitment
- Section B : Information from the iCCM Child Form on the day of recruitment.

Procedure to fill out Form 03 – Initial Visit Extraction Form

Header

1. Fill out the date when Form 03 – Initial Visit Extraction Form is filled out, the ID code of the data collector, the code of the health center.
2. Fill out the Case ID of the child, the name of the child and the name of the caregiver.
3. **The prior Case ID of the child :** As this study will be conducted over the course of 12 months, it is possible that a child gets a simple fever more than one time during the study period and participates in the study more than one time. The data collector verifies whether the child has participated in the study before and has received a Case ID previously. If the child has received a Case ID code before, fill out this previous Case ID code in the boxes. Write one digit per box. If the child has not received a Case ID previously (this is the first time the child participates in this study), write « 999999 » in the boxes.

Section A – Information from the CHW register on the day of recruitment

In this section, the data collector records information about the child that he extracts from the CHW register. The objective of this section is to write the age and sex of the child, and to verify the eligibility of the child for this study. The information that is filled out in this section is copied from the CHW

register by the data collector.

1. Record : the **date** of the first visit of the child to the CHW. This is the date when the CHW has determined the eligibility of the child for this study. The date of this visit can be found in the CHW register. Write the date in the boxes. Use the two boxes on the left to Record the day. Write the month in the two boxes in the middle, and Record the year in the two boxes on the right. Use two digits to Record the day, two digits to Record the month, and two digits to Record the year. For example: write 3 July 2016 as : 03 07 16.
2. Record: the **age** of the child. To Record the age of the child: write the years and months in the boxes. Use numbers 0 – 5 to write the number of years of the child. Use the numbers 00 – 11 to write the number of months of the child. For example: If the child is 2 years and 3 months, write: « 2 » in the box for the number of years, and write « 03 » in the boxes for the number of months.
3. Record : the **sex** of the child. If the child is male, write « M ». Write « F » if the child is female.
4. Record whether **cough/cold** is recorded in the CHW register as a complaint. If cough/cold is not recorded as a complaint, write « 0 ». Write « 1 » if cough/cold is recorded as a complaint in the CHW register. If there is no information in the CHW register, write « 9 » on the form.
5. Verify the eligibility of the child for this study: **Was the child referred for care?** If no, write « 0 » and continue to question 5b. If yes, write « 1 » and stop filling out the form because the child is not eligible for this study.
6. Verify the eligibility of the child for this study: **Was the RDT negative?** If no, write « 0 » and stop filling out the form because the child is not eligible for this study. If yes, write « 1 » and continue to question 5c.
7. Verify the eligibility of the child for this study: **Does the child have pneumonia?** If no, write « 0 » and continue to question 5d. If yes, write « 1 » and stop filling out the form because the child is not eligible for this study.
8. Verify the eligibility of the child for this study: **Does the child have diarrhea?** If no, write « 0 » and continue to question 5e. If yes, write « 1 » and stop filling out the form because the child is not eligible for this study.
9. Verify the eligibility of the child for this study: **Does the child have any danger signs?** If no, write « 0 » and continue to question 5f. If yes, write « 1 » and stop filling out the form because the child is not eligible for this study.
10. Verify the eligibility of the child for this study: **Does the child have fever?** If no, write « 0 » and stop filling out the form because the child is not eligible for this study. If yes, write « 1 » and continue.
11. Verify : **Is the iCCM Child Form corresponding to the register date available?** Verify if the date of the examination by the CHW that is written in the CHW register is the same date as is written on the iCCM Child Form of the child. If the date is not the same on both documents, write « 0 » and stop filling out the form. We will consider this child as 'not eligible' for this study as it will be impossible to calculate the dates of the Day 7, Day 14, Day 28 and Day 30 visits if there is no clarity about the date of the first visit of the child to the CHW. If the date that is written in the CHW register and the iCCM Child Form of the child are the same, write « 1 » in the box and continue to Section B.

Section B – Information from the iCCM Child Form on the day of enrollment.

In this section, the data collector Records information about the child that he extracts from the iCCM Child Form of the child. The iCCM Child Form is filled out by the CHW during every visit of the child to the CHW. To fill out this section of Form 03, the data collector uses the iCCM Child Form of the first visit of the child to the CHW (the visit during which the CHW determined the eligibility of the child for this study). The information that is filled out in this section is copied from the iCCM Child Form by the data collector.

1. Record : **Is fever listed on the iCCM Child Form as presenting complaint ?** If fever is not recorded as a presenting complaint on the iCCM Child Form, write « 0 » in the box and continue to question 3. If fever is recorded as a presenting complaint on the iCCM Child Form, write « 1 » in the box and continue to question 2. If there is no information on the iCCM Child Form, write « 9 » in the box.
2. Record : **How many days has the child had fever ?** Write the number of days that were reported by the caregiver in the box.
3. Record : **Is diarrhea listed on the iCCM Child Form as presenting complaint ?** If diarrhea is not recorded as a presenting complaint on the iCCM Child Form, write « 0 » in the box and continue to question 5. If diarrhea is recorded as a presenting complaint on the iCCM Child Form, write « 1 » in the box and continue to question 4. If there is no information on the iCCM Child Form, write « 9 » in the box.
4. Record : **How many days has the child had diarrhea ?** Write the number of days that were reported by the caregiver in the box.
5. Record : **Is cough listed on the iCCM Child Form as presenting complaint ?** If cough is not recorded as a presenting complaint on the iCCM Child Form, write « 0 » in the box and continue to question 7. If cough is recorded as a presenting complaint on the iCCM Child Form, write « 1 » in the box and continue to question 6. If there is no information on the iCCM Child Form, write « 9 » in the box.
6. Record : **How many days has the child had cough ?** Write the number of days that were reported by the caregiver in the box.
7. Record : **Other listed complaint/sign** ? If there are no other complaints or signs recorded on the iCCM Child Form, write « 0 » in the box and continue to question 8. If there are other complaints or signs recorded on the iCCM Child Form, write « 1 » in the box and continue to question 7a. If there is no information on the iCCM Child Form, write « 9 » in the box.
8. Record : **Specify other complaint** ___________. If there are other complaints or signs that are written on the iCCM Child Form, copy them here. If there are no other complaints or signs recorded on the iCCM Child Form, write « RAS » (Rien A Spécifier).
9. Record : **Specify other complaint** ___________. If there are other complaints or signs that are written on the iCCM Child Form, copy them here. If there are no other complaints or signs recorded on the iCCM Child Form, write « RAS » (Rien A Spécifier).
10. Record : **MUAC classification ?** Write the MUAC classification (that is measured by the CHW during the first visit of the child) in the box. If the classification is green, write « 1 » in the box. Write « 2 » for yellow, and « 3 » if the MUAC classification is red. If there is no information

regarding the MUAC classification on the iCCM Child Form, write « 9 » in the box.

11. Record : **Respiratory rate** that is recorded on the iCCM Child Form. Write one digit per box. If the respiratory rate is not recorded on the iCCM Child Form, record « 00 ». This question asks you to note down the respiratory rate of the child that was measured during the first visit of the child to the CHW.
12. Record : **Possibility 1 – Return visit was made.** In section 13 of the iCCM Child Form, the CHW checks the box of possibility 1 (return visit was made of the child to the CHW after 2 days) or the CHW checks the box of possibility 2 (return visit was not made of the child to the CHW after 2 days). If, on the iCCM Child Form, the box of possibility 1 – Return visit was made is blanc, write « 0 » and continue to question 10. If the box of possibility 1 – Return visit was made is checked, write « 1 » and continue to question 9a.
13. Record : **What was the mother's reported reason for the return visit?** In section 13A of the iCCM Child Form, you can find the reason why the caregiver and child came back for a return visit to the CHW. If the caregiver came back after 2 days as told by the CHW, write « 1 » in the box. Write « 2 » in the box if the caregiver returned to the CHW because the child's illness became worse. If the caregiver returned to the CHW with the child because of adverse effects, write « 3 » in the box. If there is no information on the iCCM Child Form, write « 9 ».
14. Record : **Did the mother report child's condition worsened ?** In section 13B of the iCCM Child Form, you can find the information to answer this question. If no, write « 0 » in the box, write « 1 » in the box if yes. If there is no information on the iCCM Child Form, write « 9 » in the box.
15. Record : **The child has a new complaint?** In section 13C of the iCCM Child Form, you can find the information to answer this question. If no, write « 0 » in the box, write « 1 » in the box if yes. If there is no information on the iCCM Child Form, write « 9 » in the box.
16. Record : **Is the child incapable of drinking/eating or breastfeeding?** In section 13D of the iCCM Child Form, you can find the information to answer this question. If no, write « 0 » in the box, write « 1 » in the box if yes. If there is no information on the iCCM Child Form, write « 9 » in the box.
17. Record : **Does the child vomit everything he consumes?** In section 13D of the iCCM Child Form, you can find the information to answer this question. If no, write « 0 » in the box, write « 1 » in the box if yes. If there is no information on the iCCM Child Form, write « 9 » in the box.
18. Record : **Did the child have convulsions or is convulsing now?** In section 13D of the iCCM Child Form, you can find the information to answer this question. If no, write « 0 » in the box, write « 1 » in the box if yes. If there is no information on the iCCM Child Form, write « 9 » in the box.
19. Record : **Is the child unconscious or unresponsive?** In section 13D of the iCCM Child Form, you can find the information to answer this question. If no, write « 0 » in the box, write « 1 » in the box if yes. If there is no information on the iCCM Child Form, write « 9 » in the box.
20. Record : **Does the child have palmar (or conjunctival) pallor?** In section 13D of the iCCM Child Form, you can find the information to answer this question. If no, write « 0 » in the box, write « 1 » in the box if yes. If there is no information on the iCCM Child Form, write « 9 » in the box.
21. Record : **Severe chest indrawing?** In section 13D of the iCCM Child Form, you can find the information to answer this question. If no, write « 0 » in the box, write « 1 » in the box if yes. If there is no information on the iCCM Child Form, write « 9 » in the box.

22. Record : **Persistent fever despite treatment?** In section 13D of the iCCM Child Form, you can find the information to answer this question. If no, write « 0 » in the box, write « 1 » in the box if yes. If there is no information on the iCCM Child Form, write « 9 » in the box.
23. Record : **Generalized rash/skin pustules?** In section 13D of the iCCM Child Form, you can find the information to answer this question. If no, write « 0 » in the box, write « 1 » in the box if yes. If there is no information on the iCCM Child Form, write « 9 » in the box.
24. Record : **Signs of deshydratation?** In section 13D of the iCCM Child Form, you can find the information to answer this question. If no, write « 0 » in the box, write « 1 » in the box if yes. If there is no information on the iCCM Child Form, write « 9 » in the box.
25. Record : **Blood in stool?** In section 13D of the iCCM Child Form, you can find the information to answer this question. If no, write « 0 » in the box, write « 1 » in the box if yes. If there is no information on the iCCM Child Form, write « 9 » in the box.
26. Record : **Other abnormal sign/symptom?** In section 13D of the iCCM Child Form, you can find the information to answer this question. If no, write « 0 » in the box, write « 1 » in the box if yes. If there is no information on the iCCM Child Form, write « 9 » in the box.
27. Record : **Record the respiratory rate on the iCCM Child Form**. Write one digit per box. If the respiratory rate is not recorded, write « 00 ».
28. Record : **Malaria RDT result ?** If there is no result recorded of a RDT (blanc), write « 0 » in the box. If the result of the RDT is positive, write « 1 » in the box. If the result of the RDT is negative, write « 2 » in the box.
29. Record : **Possibility 2 – No return visit.** In section 13 of the iCCM Child Form the CHW checks the box of possibility 2 (the caregiver did not return with the child to the CHW after 2 days). If, on the iCCM Child Form, the box of possibility 2 – No return visit is blanc, write « 0 ». If the box of possibility 2 – No return visit is checked, write « 1 ».
30. Verify the CHW register to answer the following question : **How many other rows are in the register for this child, after date of enrollment row, and up to today ?** The objective of this question is to know whether the child has come back to the CHW more than one time between the first visit (during which the eligibility of the child for this study was determined) and the house visit of the child on Day 7. For every additional visit during this period the CHW fills out a new iCCM Child Form. And for every additional visit during this period the CHW fills out a new line in the CHW register. Count the number of lines in the CHW register that are filled out for this child. Write « 0 » if there are no other lines filled out for this child in the CHW register, except for the line that was filled out during the first visit of the child to the CHW. If the child has visited the CHW one additional time, and the CHW has filled out one additional line in the CHW register, write « 1 » in the box and fill out Form 04 – Return Visit Extraction Form. Write « 2 » in the box if there have been two additional visits of the child to the CHW and if the CHW has filled out two additional lines in the CHW register. Now fill out Form 04 – Return Visit Extraction Form for every additional visit of the child to the CHW. Write « 3 » in the box if there have been three additional visits of the child to the CHW and if the child has filled out three additional lines in the CHW register. Now fill out Form 04 – Return Visit Extraction Form for every additional visit of the child to the CHW.

Form 04 – Return Visit Extraction Form

Form 04 – Return Visit Extraction Form is filled out for every child that is eligible for this study (The child has a simple fever, RDT negative, without diarrhea, without pneumonia, without any danger signs, the child is not referred for care to a health center) and that returns to the CHW between his first visit (Day 1) and the house visit of the child by the CHW and data collector on Day 7. The return visit can occur because the CHW asked the child to return after two days, or it can be a spontaneous return visit because the health of the child became worse, the child has new complaints/signs, etc. Form 04 is used by the data collector to extract information from the CHW register and the iCCM Child Form that is filled out by the CHW during the return visit. During every return visit of the child after the first visit to the CHW, the CHW fills out his register and a new iCCM Child Form. In the CHW register and on the iCCM Child Form information regarding the examination by the CHW can be found, including the complaints of the child, different measures (MUAC, respiratory rate), the result of the RDT (if done), information about any danger signs and warning signs and the treatment of the child by the CHW.

Form 04 – Return Visit Extraction Form is filled out by the data collector at the house of the CHW on the same day as the house visit of the child is done on Day 7.

Elements of Form 04 – Return Visit Extraction Form

Header : The following elements are included in the header of the form : the date when the form is filled out by the data collector, the data collector's ID code, the code of the health center, the case ID for the child, the name of the child, the name of the caregiver.

Sections : This form has 3 sections, each section is explained below. The sections of this form are:

- Section A : Information from CHW register on the day of the return visit of the child
- Section B : Information from the iCCM Child Form on the day of the return visit of the child
- Section C : Information from the iCCM Child Form on the day of the return visit of the child

Procedure for filling out Form 04 – Return Visit Extraction Form

Header

1. Fill out the date when Form 04 – Return Visit Extraction Form is filled out, the ID code of the data collector, the code of the health center.
2. Fill out the Case ID of the child, the name of the child and the name of the caregiver.

Section A : Information from CHW register on the day of the return visit of the child

In this section, we want to learn more about the health of the child based on the CHW register. The data collector uses the line in the CHW register that is filled out by the CHW during the return visit of the child.

1. Record : the **date** of the return visit of the child to the CHW. The date of this visit can be found in the CHW register. Write the date in the boxes. Use the two boxes on the left to record the day. Write the month in the two boxes in the middle, and record the year in the two boxes on the right. Use two digits to Record the day, two digits to record the month, and two digits to record the year. For example: write 3 July 2016 as : 03 07 16.
2. Record : whether the child has had **a consultation préscolaire (CPS)**. You can find the answer to

this question in the CHW register. If no, write « 0 » in the box. If yes, write « 1 » in the box. If there is no information in the CHW register, write « 9 » in the box.

3. Record: whether the child is in order with **Vitamin A**. You can find the answer to this question in the CHW register. If no, write « 0 » in the box. If yes, write « 1 » in the box. If there is no information in the CHW register, write « 9 » in the box.
4. Record : whether the child is in order with **vaccinations.** You can find the answer to this question in the CHW register. If no, write « 0 » in the box. If yes, write « 1 » in the box. If there is no information in the CHW register, write « 9 » in the box.
5. Record : whether there are any **danger signs** recorded in the CHW register. You can find the answer to this question in the CHW register. If no, write « 0 » in the box. If yes, write « 1 » in the box. If there is no information in the CHW register, write « 9 » in the box.
6. Record : whether **cough/cold** is recorded as a complaint in the CHW register. You can find the answer to this question in the CHW register. If no, write « 0 » in the box. If yes, write « 1 » in the box. If there is no information in the CHW register, write « 9 » in the box.
7. Record : whether **fever** is recorded as a complaint in the CHW register. You can find the answer to this question in the CHW register. If no, write « 0 » in the box. If yes, write « 1 » in the box. If there is no information in the CHW register, write « 9 » in the box.
8. Record : whether **diarrhea** is Recorded as a complaint in the CHW register. You can find the answer to this question in the CHW register. If no, write « 0 » in the box. If yes, write « 1 » in the box. If there is no information in the CHW register, write « 9 » in the box.
9. Record : whether **fast breathing** is Recorded in the CHW register. You can find the answer to this question in the CHW register. If no, write « 0 » in the box. If yes, write « 1 » in the box. If there is no information in the CHW register, write « 9 » in the box.
10. Record: whether the **RDT status** of the child is recorded in the CHW register. You can find the answer to this question in the CHW register. If no, write « 0 » in the box. If yes, write « 1 » in the box. If there is no information in the CHW register, write « 9 » in the box.
11. Record : whether the **MUAC classification** of the child is Recorded in the CHW register. You can find the answer to this question in the CHW register. If no, write « 0 » in the box. If yes, write « 1 » in the box. If there is no information in the CHW register, write « 9 » in the box.
12. Record : whether **cough/cold** is Recorded in the CHW register. You can find the answer to this question in the CHW register. If no, write « 0 » in the box. If yes, write « 1 » in the box. If there is no information in the CHW register, write « 9 » in the box.
13. Record : whether **pneumonia** is Recorded in the CHW register. You can find the answer to this question in the CHW register. If no, write « 0 » in the box. If yes, write « 1 » in the box. If there is no information in the CHW register, write « 9 » in the box.
14. Record : whether **malaria (confirmed – RDT+)** is Recorded in the CHW register. You can find the answer to this question in the CHW register. If malaria is not confirmed, the result of the RDT is not positive or the result of the RDT is not available (RDT is not done), write « 0 » in the box. If malaria is confirmed by a positive RDT, write « 1 » in the box. If there is no information in the CHW register, write « 9 » in the box.
15. Record : whether **fever (RDT -)** is recorded in the CHW register. You can find the answer to this question in the CHW register. If no (the child does not have a fever and the result of the RDT is

negative), write « 0 » in the box. If yes (the child has a fever and the result of the RDT is negative), write « 1 » in the box. If there is no information in the CHW register, write « 9 » in the box.

16. Record : whether **malaria (suspected)** is recorded in the CHW register. You can find the answer to this question in the CHW register. If no (the result of the RDT is available and is either positive or negative), write « 0 » in the box. If yes (the result of the RDT is not available), write « 1 » in the box. If there is no information in the CHW register, write « 9 » in the box.
17. Record : whether **diarrhea** is recorded in the CHW register. You can find the answer to this question in the CHW register. If no, write « 0 » in the box. If yes, write « 1 » in the box. If there is no information in the CHW register, write « 9 » in the box.
18. Record : whether **moderate malnutrition** is recorded in the CHW register. You can find the answer to this question in the CHW register. If no, write « 0 » in the box. If yes, write « 1 » in the box. If there is no information in the CHW register, write « 9 » in the box.
19. Record : whether **severe malnutrition** is recorded in the CHW register. You can find the answer to this question in the CHW register. If no, write « 0 » in the box. If yes, write « 1 » in the box. If there is no information in the CHW register, write « 9 » in the box.
20. Record : whether **Amoxicillin** is recorded in the CHW register as treatment. You can find the answer to this question in the CHW register. If no, write « 0 » in the box. If yes, write « 1 » in the box. If there is no information in the CHW register, write « 9 » in the box.
21. Record : whether **ACT (for a child with a positive RDT result**) is recorded in the CHW register as treatment. You can find the answer to this question in the CHW register. If no, write « 0 » in the box. If yes, write « 1 » in the box. If there is no information in the CHW register, write « 9 » in the box.
22. Record : whether **ACT (for a suspected case of malaria)** is recorded in the CHW register as treatment. You can find the answer to this question in the CHW register. If no, write « 0 » in the box. If yes, write « 1 » in the box. If there is no information in the CHW register, write « 9 » in the box.
23. Record : whether **ORS/Zinc** is recorded in the CHW register as treatment. You can find the answer to this question in the CHW register. If no, write « 0 » in the box. If yes, write « 1 » in the box. If there is no information in the CHW register, write « 9 » in the box.
24. Record : whether **the child is referred for care**. You can find the answer to this question in the CHW register. If no, write « 0 » in the box. If yes, write « 1 » in the box. If there is no information in the CHW register, write « 9 » in the box.
25. Verify : **Is the iCCM Child Form corresponding to the register date available?** Verify if the date of the examination by the CHW that is written in the CHW register is the same date as is written on the iCCM Child Form of the child. If the date is not the same on both documents, write « 0 » and stop filling out the form. We will consider this child as 'not eligible' for this study as it will be impossible to calculate the dates of the Day 7, Day 14, Day 28 and Day 30 visits if there is no clarity about the date of the first visit of the child to the CHW. If the date that is written in the CHW register and the iCCM Child Form of the child are the same, write « 1 » in the box and continue to Section B.

Section B - Information from iCCM Child Form corresponding to the follow up register row - Information from the iCCM Child Form on the day of the return visit of the child

In this section, the data collector Records information about the child that he extracts from the iCCM Child Form of the child. The iCCM Child Form is filled out by the CHW during every visit of the child to the CHW. To fill out this section of Form 04, the data collector uses the iCCM Child Form of the return visit of the child to the CHW. The objective of this section is to extract information about the health of the child during the return visit. The information that is filled out in this section is copied from the iCCM Child Form by the data collector.

1. Record : **Is fever listed on the iCCM Child Form as presenting complaint ?** If fever is not recorded as a presenting complaint on the iCCM Child Form, write « 0 » in the box and continue to question 3. If fever is recorded as a presenting complaint on the iCCM Child Form, write « 1 » in the box and continue to question 2. If there is no information on the iCCM Child Form, write « 9 » in the box.
2. Record : **How many days has the child had fever ?** Write the number of days that were reported by the caregiver in the box.
3. Record : **Is diarrhea listed on the iCCM Child Form as presenting complaint ?** If diarrhea is not recorded as a presenting complaint on the iCCM Child Form, write « 0 » in the box and continue to question 5. If diarrhea is recorded as a presenting complaint on the iCCM Child Form, write « 1 » in the box and continue to question 4. If there is no information on the iCCM Child Form, write « 9 » in the box.
4. Record : **How many days has the child had diarrhea ?** Write the number of days that were reported by the caregiver in the box.
5. Record : **Is cough listed on the iCCM Child Form as presenting complaint ?** If cough is not recorded as a presenting complaint on the iCCM Child Form, write « 0 » in the box and continue to question 7. If cough is recorded as a presenting complaint on the iCCM Child Form, write « 1 » in the box and continue to question 6. If there is no information on the iCCM Child Form, write « 9 » in the box.
6. Record : **How many days has the child had cough ?** Write the number of days that were reported by the caregiver in the box.
7. Record : **Other listed complaint/sign** ? If there are no other complaints or signs Recorded on the iCCM Child Form, write « 0 » in the box and continue to question 8. If there are other complaints or signs Recorded on the iCCM Child Form, write « 1 » in the box and continue to question 7a. If there is no information on the iCCM Child Form, write « 9 » in the box.
8. Record : **Specify other complaint** ___________. If there are other complaints or signs that are written on the iCCM Child Form, copy them here. If there are no other complaints or signs Recorded on the iCCM Child Form, write « RAS » (Rien A Spécifier).
9. Record : **Specify other complaint** ___________. If there are other complaints or signs that are written on the iCCM Child Form, copy them here. If there are no other complaints or signs Recorded on the iCCM Child Form, write « RAS » (Rien A Spécifier).
10. Record : **MUAC classification ?** Write the MUAC classification (that is measured by the CHW during the first visit of the child) in the box. If the classification is green, write « 1 » in the box.

Write « 2 » for yellow, and « 3 » if the MUAC classification is red. If there is no information regarding the MUAC classification on the iCCM Child Form, write « 9 » in the box.

11. Record : **Is the child incapable of drinking/eating or breastfeeding?** In section 3A of the iCCM Child Form, you can find the information to answer this question. If no, write « 0 » in the box, write « 1 » in the box if yes. If there is no information on the iCCM Child Form, write « 9 » in the box.
12. Record : **Does the child vomit everything he consumes?** In section 3A of the iCCM Child Form, you can find the information to answer this question. If no, write « 0 » in the box, write « 1 » in the box if yes. If there is no information on the iCCM Child Form, write « 9 » in the box.
13. Record : **Did the child have convulsions or is convulsing now?** In section 3A of the iCCM Child Form, you can find the information to answer this question. If no, write « 0 » in the box, write « 1 » in the box if yes. If there is no information on the iCCM Child Form, write « 9 » in the box.
14. Record : **Is the child unconscious or unresponsive?** In section 3A of the iCCM Child Form, you can find the information to answer this question. If no, write « 0 » in the box, write « 1 » in the box if yes. If there is no information on the iCCM Child Form, write « 9 » in the box.
15. Record : **Is MUAC measurement RED?** In section 3B of the iCCM Child Form, you can find the information to answer this question. If no, write « 0 » in the box, write « 1 » in the box if yes. If there is no information on the iCCM Child Form, write « 9 » in the box.
16. Record : **Is illness more than 14 days, or fever more than 7 days?** In section 3B of the iCCM Child Form, you can find the information to answer this question. If no, write « 0 » in the box, write « 1 » in the box if yes. If there is no information on the iCCM Child Form, write « 9 » in the box.
17. Record : **Does the child have difficulty breathing while wheezing?** In section 3B of the iCCM Child Form, you can find the information to answer this question. If no, write « 0 » in the box, write « 1 » in the box if yes. If there is no information on the iCCM Child Form, write « 9 » in the box.
18. Record : **Does the child have palmar (or conjunctival) pallor?** In section 3B of the iCCM Child Form, you can find the information to answer this question. If no, write « 0 » in the box, write « 1 » in the box if yes. If there is no information on the iCCM Child Form, write « 9 » in the box.
19. Record : **Is the child often sick?** In section 3B of the iCCM Child Form, you can find the information to answer this question. If no, write « 0 » in the box, write « 1 » in the box if yes. If there is no information on the iCCM Child Form, write « 9 » in the box.
20. Record : **Is the child very weak?** In section 3B of the iCCM Child Form, you can find the information to answer this question. If no, write « 0 » in the box, write « 1 » in the box if yes. If there is no information on the iCCM Child Form, write « 9 » in the box.
21. Record : **Is the child becoming sicker despite care at home?** In section 3B of the iCCM Child Form, you can find the information to answer this question. If no, write « 0 » in the box, write « 1 » in the box if yes. If there is no information on the iCCM Child Form, write « 9 » in the box.
22. Record : **Does the child have visible signs of wasting?** In section 3C of the iCCM Child Form, you can find the information to answer this question. If no, write « 0 » in the box, write « 1 » in the box if yes. If there is no information on the iCCM Child Form, write « 9 » in the box.
23. Record : **Does the child have edema of lower limbs?** In section 3C of the iCCM Child Form, you

can find the information to answer this question. If no, write « 0 » in the box, write « 1 » in the box if yes. If there is no information on the iCCM Child Form, write « 9 » in the box.

24. Record : **Does the child have diarrhea with dehydration or blood in stools**? In section 3D of the iCCM Child Form, you can find the information to answer this question. If no, write « 0 » in the box, write « 1 » in the box if yes. If there is no information on the iCCM Child Form, write « 9 » in the box.
25. Record : **Does the child have a fever with generalized skin condition/rash/measles?** In section 3D of the iCCM Child Form, you can find the information to answer this question. If no, write « 0 » in the box, write « 1 » in the box if yes. If there is no information on the iCCM Child Form, write « 9 » in the box.
26. Record : **Record the respiratory rate on the iCCM Child Form**. Write one digit per box. If the respiratory rate is not recorded, write « 00 » in the boxes.
27. Record : Does the child have a fever (hot to touch or fever within two days)? If no, write « 0 » in the box and continue to Section C. Write « 1 » in the box if yes and continue to question 10a. If there is no information on the iCCM Child Form, write « 9 » in the box.
28. If the child has a fever: Record : **Malaria RDT result ?** If there is no result recorded of a RDT (blanc), write « 0 » in the box. If the result of the RDT is positive, write « 1 » in the box. If the result of the RDT is negative, write « 2 » in the box.

<u>Section C – Information from the iCCM Child Form corresponding to the follow up register row (Back Page) – Information from the iCCM Child Form on the day of the return visit of the child</u>

1. Record : **Possibility 1 – Return visit was made.** In section 13 of the iCCM Child Form, the CHW checks the box of possibility 1 (return visit was made of the child to the CHW after 2 days) or the CHW checks the box of possibility 2 (return visit was not made of the child to the CHW after 2 days). If, on the iCCM Child Form, the box of possibility 1 – Return visit was made is blanc, write « 0 » and continue to question 2. If the box of possibility 1 – Return visit was made is checked, write « 1 » and continue to question 1a.
2. Record : **What was the mother's reported reason for the return visit?** In section 13A of the iCCM Child Form, you can find the reason why the caregiver and child came back for a return visit to the CHW. If the caregiver came back after 2 days as told by the CHW, write « 1 » in the box. Write « 2 » in the box if the caregiver returned to the CHW because the child's illness became worse. If the caregiver returned to the CHW with the child because of adverse effects, write « 3 » in the box. If there is no information on the iCCM Child Form, write « 9 ».
3. Record : **Did the mother report child's condition worsened ?** In section 13B of the iCCM Child Form, you can find the information to answer this question. If no, write « 0 » in the box, write « 1 » in the box if yes. If there is no information on the iCCM Child Form, write « 9 » in the box.
4. Record : **The child has a new complaint?** In section 13C of the iCCM Child Form, you can find the information to answer this question. If no, write « 0 » in the box, write « 1 » in the box if yes. If there is no information on the iCCM Child Form, write « 9 » in the box.
5. Record : **Is the child incapable of drinking/eating or breastfeeding?** In section 13D of the iCCM Child Form, you can find the information to answer this question. If no, write « 0 » in the box,

write « 1 » in the box if yes. If there is no information on the iCCM Child Form, write « 9 » in the box.

6. Record : **Does the child vomit everything he consumes?** In section 13D of the iCCM Child Form, you can find the information to answer this question. If no, write « 0 » in the box, write « 1 » in the box if yes. If there is no information on the iCCM Child Form, write « 9 » in the box.
7. Record : **Did the child have convulsions or is convulsing now?** In section 13D of the iCCM Child Form, you can find the information to answer this question. If no, write « 0 » in the box, write « 1 » in the box if yes. If there is no information on the iCCM Child Form, write « 9 » in the box.
8. Record : **Is the child unconscious or very weak?** In section 13D of the iCCM Child Form, you can find the information to answer this question. If no, write « 0 » in the box, write « 1 » in the box if yes. If there is no information on the iCCM Child Form, write « 9 » in the box.
9. Record : **Does the child have palmar (or conjunctival) pallor?** In section 13D of the iCCM Child Form, you can find the information to answer this question. If no, write « 0 » in the box, write « 1 » in the box if yes. If there is no information on the iCCM Child Form, write « 9 » in the box.
10. Record : **Severe chest indrawing?** In section 13D of the iCCM Child Form, you can find the information to answer this question. If no, write « 0 » in the box, write « 1 » in the box if yes. If there is no information on the iCCM Child Form, write « 9 » in the box.
11. Record : **Child became sicker?** In section 13D of the iCCM Child Form, you can find the information to answer this question. If no, write « 0 » in the box, write « 1 » in the box if yes. If there is no information on the iCCM Child Form, write « 9 » in the box.
12. Record : **Persistent fever despite treatment?** In section 13D of the iCCM Child Form, you can find the information to answer this question. If no, write « 0 » in the box, write « 1 » in the box if yes. If there is no information on the iCCM Child Form, write « 9 » in the box.
13. Record : **Generalized rash/skin pustules?** In section 13D of the iCCM Child Form, you can find the information to answer this question. If no, write « 0 » in the box, write « 1 » in the box if yes. If there is no information on the iCCM Child Form, write « 9 » in the box.
14. Record : **Signs of deshydratation?** In section 13D of the iCCM Child Form, you can find the information to answer this question. If no, write « 0 » in the box, write « 1 » in the box if yes. If there is no information on the iCCM Child Form, write « 9 » in the box.
15. Record : **Blood in stool?** In section 13D of the iCCM Child Form, you can find the information to answer this question. If no, write « 0 » in the box, write « 1 » in the box if yes. If there is no information on the iCCM Child Form, write « 9 » in the box.
16. Record : **Loose, watery stools?** In section 13D of the iCCM Child Form, you can find the information to answer this question. If no, write « 0 » in the box, write « 1 » in the box if yes. If there is no information on the iCCM Child Form, write « 9 » in the box.
17. Record : **Other abnormal sign/symptom?** In section 13D of the iCCM Child Form, you can find the information to answer this question. If no, write « 0 » in the box, write « 1 » in the box if yes. If there is no information on the iCCM Child Form, write « 9 » in the box.
18. Record : **Record the respiratory rate on the iCCM Child Form**. Write one digit per box. If the respiratory rate is not recorded, write « 00 » in the boxes.
19. Record : **Malaria RDT result ?** If there is no result recorded of a RDT (blanc), write « 0 » in the box. If the result of the RDT is positive, write « 1 » in the box. If the result of the RDT is negative,

write « 2 » in the box.

20. Record : **Possibility 2 – No return visit.** In section 13 of the iCCM Child Form the CHW checks the box of possibility 2 (the caregiver did not return with the child to the CHW after 2 days). If, on the iCCM Child Form, the box of possibility 2 – No return visit is blanc, write « 0 ». If the box of possibility 2 – No return visit is checked, write « 1 ».

Form 05 – Consent Form

This consent form must be read before collecting any information during the house visit of the child on Day 7. The child will not be visited at his house by the data collector and CHW if the caregiver does not give permission for the house visit during the first visit of the child to the CHW (during this visit, the CHW determines the eligibility of the child, and reads the recruitment statement to the caregiver).

Before starting the examination of the child by the CHW and the data collector during the house visit of the child on Day 7, the consent statement must be read out loud by the data collector. The consent statement describes the main objective of the study and the procedures that will be carried during each of the house visits of the child (on Day 7, Day 14, and Day 28). It explains that we are interested in learning more about children who have a simple fever and how their health develops. It describes that we will follow the child with simple fever over the course of 30 days. It explains that the data collector and the CHW will visit the child at least one time during the 30 day follow-up period, and the duration of each visit. It also explains that all the information will be kept confidential. Voluntariness is explained, and the contact details of the Research Manager are given. Every data collector will have one consent form for every child that is eligible for this study. The data collector writes on the consent form whether the caregiver gave his/her consent for participation of the child in this study.

Procedure for conducting consent for child's participation in this study :

1. Read the oral consent script out loud. You will find the oral consent script on the consent form.
2. Answer any questions that the caregiver might have about the information provided in the script.
3. Ask : «do you agree to participate in this study ? »
4. If the caregiver refuses participation, record a «0» in the box for consent on Form 05 and Form 06, and thank him/her for his/her time. Do not fill out the rest of Form 06.

e. If the caregiver agrees to participate in this study, record a «1» in the box for consent on Form 05.

F. If the caregiver gives his/her consent, record a «1» in the box for consent on Form 06, and continue to fill out Form 06.

Form 06 – Day 7 Visit Form

Every child that is eligible for this study (the child has a simple fever – RDT negative, without diarrhea, without pneumonia, without any danger signs, the child is not referred for care to the health center) will be visit at his/her house by the data collector and the CHW if the caregiver gives permission for this visit. The first house visit is planned by the data collector and CHW 6 days after the first visit of the child to the CHW (the visit when the eligibility of the child was determined by the CHW), on Day 7. If Day 7 falls on a Sunday, the data collector plans a day before, namely on Saturday (Day 6). The data collector fills out Form 06 – Day 7 Assessment Form during this house visit. On Form 06 – Day 7 Assessment Form we collect information about the health of the child. The data collector takes a several measurements (temperature, MUAC, respiratory rate) that are recorded on Form 06. The CHW conducts an examination of the child to get additional information about the child's health.

Elements of Form 06 – Day 7 Assessment Form

Header : The following elements are included in the header of the form : the date when the form is filled out by the data collector, the data collector's ID code, the code of the health center, the case ID for the child, the name of the child, the name of the caregiver.

Sections : This form has 4 sections, each section is explained below. The sections of this form are

- Section A : Data collector's measures
- Section B : Results from CHW assessment
- Section C : Classification of the child after assessment
- Section D : Treatment / action taken by CHW

Procedure to fill out Form 06 – Day 7 Assessment Form

Header :

1. Fill out the date when Form 06 – Day 7 Assessment Form is filled out, the ID code of the data collector, the code of the health center.
2. Fill out the Case ID of the child, the name of the child and the name of the caregiver.
3. **Consent :** record the consent of the caregiver on the form. If the caregiver refuses participation in the study, and does not give consent, write « 0 » in the box and stop filling out Form 06. If the caregiver agrees to participation in the study, and gives consent, write « 1 » in the box and continue filling out the form.
4. **Record child status :** record the vital status of the child in the box. Record « 1 » if the child is alive and present during the house visit on Day 7, and continue filling out Form 06. Record « 2 » if the child died, « 3 » if the child is hospitalized and is never assessed, and « 4 » if the child is lost to follow up (the child is not present). If the child is not present during the planned house visit on Day 7, return the next day to the house of the child to complete Form 06. If « 2 », « 3 », or « 4 » were filled out stop filling out Form 06, and continue with Form 07 – Care-seeking and Demographic Form.

Section A – Data Collector's Measures

In this section, 3 different measures are taken by the data collector. The measures of the child that are taken by the data collector are a supplement to the examination of the child done by the CHW. The

CHW conducts his examination of the child as he always does (the examination of the child when he is sick and visits the CHW). In addition to this examination, the data collector measures the child's temperature, MUAC, and respiratory rate. Record the measurements in digits in the boxes.

1. Use the digital thermometer to measure the body temperature of the child. Record the temperature of the child in the boxes. Write one digit per box and record one decimal.
2. Use the MUAC measurement tool to measure the circumference of the mid-upper arm of the child to examine the nutritional state of the child. Record the circumference of the arm of the child in the boxes. Write one digit per box and record one decimal.
3. Use a stopwatch to record the respiratory rate of the child. Count the number of respiratory movements during one minute. Record the respiratory rate in the boxes. Write one digit per box.

Measuring Child's Body Temperature

1. Turn on the thermometer by pressing on the small round button adjacent to the display. Make sure the display reads "0.0".
2. Clean the thermometer with a piece of alcohol-soaked cotton.
3. Place the thermometer under the armpit of the child and hold it in place until it beeps.
4. After the thermometer beeps, remove it from the armpit of the child and record the temperature in Celsius. When recording on the form, use two digits to the left if the decimal place, and one digit to the right. For example, when the child's temperature is 37.5C, record «37.5» in the boxes.
5. Clean the thermometer with a piece of alcohol-soaked cotton. Only one temperature measurement is required during the house visit.

Measuring Child's Mid-Upper Arm Circumference (using the MUAC tool)

1. Take the left arm of the child.
2. Place the left arm of the child across his chest and ask the caregiver to keep the arm in this position.
3. Use the MUAC tool to determine the midpoint between the olecranon process and the acromion process.
4. Strap the MUAC tool around the arm of the child at the identified midpoint.
5. With the arm of the child hanging down, record the digits that are shown at the arrow in the hole of the MUAC tool (round to the nearest 1 mm).
6. When recording on the form, use two digits to the left of the decimal place, and one digit to the right.

Measuring Child's Respiratory Rate

1. Turn on the stopwatch with the button adjacent to the display. Make sure that the display reads : « 00 :00 ».
2. Start the stopwatch for one minute (60 seconds).
3. Count the respiratory movements of the child during one minute (60 seconds).
4. When recording on the form, use two digits for the respiratory rate of the child.

Section B – Results from the CHW's Assessment

In this section, the data collector records the results from the examination that is done by the CHW. The data collector is present during the examination of the child by the CHW. He follows the examination and records the results in this section. Record the responses in the boxes.

1. Record : **Does the child have fever ?** If the child does not have a fever, write « 0 » in the box and continue to question 2. If the child has a fever, write « 1 » in the box. If there is no information available, write « 9 » in the box. If the child has a fever, write the number of days that the caregiver reported the child having a fever in the box in right column, using one digit.
2. Record : **Does the child have diarrhea ?** If the child does not have diarrhea, write « 0 » in the box and continue to question 3. If the child has diarrhea, write « 1 » in the box. If there is no information available, write « 9 » in the box. If the child has diarrhea, write the number of days that the caregiver reported the child having diarrhea in the box in right column, using one digit.
3. Record : **Does the child have cough ?** If the child does not have cough, write « 0 » in the box and continue to question 4. If the child has cough, write « 1 » in the box. If there is no information available, write « 9 » in the box. If the child has cough, write the number of days that the caregiver reported the child having cough in the box in right column, using one digit.
4. Record : **Does the child have any other complaint** ? If there are no other complaints, write « 0 » in the box and continue to question 5. If there are other complaints, write « 1 » in the box and continue to question 4a. If there is no information available, write « 9 » in the box.
5. Record : **Specify other complaint ___________**. If there are other complaints, record them here. If there are no other complaints, write « RAS » (Rien A Spécifier).
6. Record : **Specify other complaint ___________**. If there are other complaints, record them here. If there are no other complaints, write « RAS » (Rien A Spécifier).
7. Record : **MUAC classification ?** Write the MUAC classification (that is measured by the CHW during the examination of the child) in the box. If the classification is green, write « 1 » in the box. Write « 2 » for yellow, and « 3 » if the MUAC classification is red. If there is no information regarding the MUAC classification, write « 9 » in the box.
8. Record : **Is the child incapable of drinking/eating or breastfeeding?** If no, write « 0 » in the box, write « 1 » in the box if yes. If there is no information available, write « 9 » in the box.
9. Record : **Does the child vomit everything he consumes?** If no, write « 0 » in the box, write « 1 » in the box if yes. If there is no information available, write « 9 » in the box.
10. Record : **Did the child have convulsions or is convulsing now?** If no, write « 0 » in the box, write « 1 » in the box if yes. If there is no information available, write « 9 » in the box.
11. Record : **Is the child unconscious or unresponsive?** If no, write « 0 » in the box, write « 1 » in the box if yes. If there is no information available, write « 9 » in the box.
12. Record : **Is MUAC measurement RED?** If no, write « 0 » in the box, write « 1 » in the box if yes. If there is no information available, write « 9 » in the box.
13. Record : **Is illness more than 14 days, or fever more than 7 days?** If no, write « 0 » in the box, write « 1 » in the box if yes. If there is no information available, write « 9 » in the box.
14. Record : **Does the child have difficulty breathing while wheezing?** If no, write « 0 » in the box, write « 1 » in the box if yes. If there is no information available, write « 9 » in the box.

15. Record : **Does the child have palmar (or conjunctival) pallor?** If no, write « 0 » in the box, write « 1 » in the box if yes. If there is no information available, write « 9 » in the box.
16. Record : **Is the child often sick?** If no, write « 0 » in the box, write « 1 » in the box if yes. If there is no information available, write « 9 » in the box.
17. Record : **Is the child very weak?** If no, write « 0 » in the box, write « 1 » in the box if yes. If there is no information available, write « 9 » in the box.
18. Record : **Is the child becoming sicker despite care at home?** If no, write « 0 » in the box, write « 1 » in the box if yes. If there is no information available, write « 9 » in the box.
19. Record : **Does the child have visible signs of wasting?** If no, write « 0 » in the box, write « 1 » in the box if yes. If there is no information available, write « 9 » in the box.
20. Record : **Does the child have edema of lower limbs?** If no, write « 0 » in the box, write « 1 » in the box if yes. If there is no information available, write « 9 » in the box.
21. Record : **Does the child have diarrhea with dehydration or blood in stools?** If no, write « 0 » in the box, write « 1 » in the box if yes. If there is no information available, write « 9 » in the box.
22. Record : **Does the child have a fever with generalized skin condition/rash?** If no, write « 0 » in the box, write « 1 » in the box if yes. If there is no information available, write « 9 » in the box.
23. Record : **Record the respiratory rate of the child as measured by the CHW**. Write one digit per box. If the respiratory rate is not recorded, write « 00 » in the boxes.
24. Record : **Does the child have a fever (hot to touch or fever within two days)?** If no, write « 0 » in the box and continue to Section C. Write « 1 » in the box if yes and continue to question 5a. If there is no information available, write « 9 » in the box.
25. If the child has a fever: Record : **What is the RDT result ?** If there is no result recorded of a RDT (RDT is not done, box is blank), write « 0 » in the box. If the result of the RDT is positive, write « 1 » in the box. If the result of the RDT is negative, write « 2 » in the box.

Section C – Classification of the Child after Assessment

In this section the classification of the child after the examination by the CHW is recorded. The CHW determines the classification of the child based on his examination. The data collector Records information about the classification on this form. This section seeks to collect information about the following classifications: malaria, diarrhea, pneumonia, moderate malnutrition, fever, and cough. Record the responses in the boxes, using one digit.

1. Record the classification of the child after examination by the CHW of **malaria**. If the child does not have malaria, record « 0 » in the box and continue to question 2. If the child has malaria, record « 1 » in the box and continue to question 1a. If there is no information available, record « 9 » in the box.
2. If the CHW determines that the child has malaria, record whether the child is a **confirmed or a presumed case of malaria**. Record « 1 » if it is a confirmed case (result of the TDR is positive). Record « 2 » if it is a presumed case (result of the RDT is not available).
3. Record the classification of the child after examination by the CHW of **diarrhea**. If the child does not have diarrhea, record « 0 » in the box. If the child has diarrhea, record « 1 » in the box. If there is no information available, record « 9 » in the box.

4. Record the classification of the child after examination by the CHW of **pneumonia**. If the child does not have pneumonia, record « 0 » in the box. If the child has pneumonia, record « 1 » in the box. If there is no information available, record « 9 » in the box.
5. Record the classification of the child after examination by the CHW of **moderate malnutrition**. If the child does not have moderate malnutrition, record « 0 » in the box. If the child has moderate malnutrition, record « 1 » in the box. If there is no information available, record « 9 » in the box.
6. Record other classifications of the child after examination by the CHW using **other (describe)**. Write other classifications on the form. If there are no other classifications after examination by the CHW, write « RAS » (Rien A Spécifier).
7. Record the classification of the child after examination by the CHW of **fever (with or without cough)**. If the child does not have fever (with or without cough), record « 0 » in the box. If the child has fever (with or without cough), record « 1 » in the box. If there is no information available, record « 9 » in the box.
8. Record the classification of the child after examination by the CHW of **cough**. If the child does not have cough, record « 0 » in the box. If the child has cough, record « 1 » in the box. If there is no information available, record « 9 » in the box.

<u>Section D – Treatment/action taken by CHW</u>

In this section information is recorded about the treatment of the child after examination by the CHW. The CHW determines the treatment of the child based on his examination of the child. The data collector records the information on this form. Record the responses in the boxes.

1. Record: **Antimalarials given?** Record whether the CHW has given any antimalarials to the child. If the CHW did not give any antimalarials to the child, record « 0 » and continue to question 2. Record « 1 » if the CHW did give any antimalarials to the child, and continue to question 1a. If there is no information available, record « 9 » and continue to question 2.
2. If the CHW gave any antimalarials, record : **Describe_______________.** Describe which type of antimalarials the CHW gave to the child. Record « RAS » (Rien A Spécifier) if the CHW gave antimalarials to the child, but there is no information available about which type of antimalarials.
3. Record: **Antibiotics given?** Record whether the CHW has given any antibiotics to the child. If the CHW did not give any antibiotics to the child, record « 0 » and continue to question 3. Record « 1 » if the CHW did give any antibiotics to the child, and continue to question 2a. If there is no information available, record « 9 » and continue to question 3.
4. If the CHW gave any antibiotics, record : **Describe_______________.** Describe which type of antibiotics the CHW gave to the child. Record « RAS » (Rien A Spécifier) if the CHW gave antibiotics to the child, but there is no information available about which type of antibiotics.
5. Record: **ORS/Zinc given?** Record whether the CHW has given any ORS/Zinc to the child. If the CHW did not give any ORS/Zinc to the child, record « 0 » and continue to question 4. Record « 1 » if the CHW did give any ORS/Zinc to the child, and continue to question 3a. If there is no information available, record « 9 » and continue to question 4.

6. If the CHW gave any ORS/Zinc, record : **Describe______________.** Describe which type of ORS/Zinc the CHW gave to the child. Record « RAS » (Rien A Spécifier) if the CHW gave ORS/Zinc to the child, but there is no information available about which type of ORS/Zinc.
7. Record : **Paracetamol given ?** Record whether the CHW has given any paracetamol to the child. If the CHW did not give any paracetamol to the child, record « 0 ». Record « 1 » if the CHW gave paracetamol to the child. If there is no information available, record « 9 » in the box.
8. Determine: **Did the child have fever, or was the child treated, or was the child referred for care to a health center?** If no (the child did not have a fever, the child was not treated by the CHW, and the child was not referred for care to a health center), record « 0 » in the box and contact the child's caregiver on Day 30 to record the vital status of the child. If yes to at least one criteria (the child has a fever, or the child is treated by the CHW, or the child was referred for care to a health center), record « 1 » in the box and plan the house visit of the child on Day 14.
9. If C6 = 0 (the child does not have fever during the Day 7 visit), ask the following question: **How many days ago did your child last have fever?** Record the answer to this question in number of days in the boxes using two digits. Record « 00 » if the child last had fever today, « 01 » to « 28 » for the numbers of days and « 99 » if the mother or caregiver does not know how many days ago the child last had fever.

Form 07 – Care-seeking and Demographic Form

The data collector fills out Form 07 – Care-seeking and Demographic Form for every child that is eligible for this study, and consent for participation has been given by the caregiver. Form 07 – Care-seeking and Demographic Form is filled out during the house visit of the child on Day 7. However, the form can also be filled out on another day if the caregiver prefers Form 07 – Care-seeking and Demographic Form to be filled out on another day (for example because the child is hospitalized or dead and this is not a good moment to interview the caregiver about care-seeking behavior and demographic characteristics). Record that Form 07 – Care-seeking and Demographic Form always has to be filled out during the 30 days of enrollment of the child in this study.

The objective of this form is to collect information about the characteristics of the household of te child. The data collector asks questions to the caregiver in Swahili and records the caregiver's responses on the form. The data collector asks the caregiver questions about the care that the child received before coming to the CHW, about the care that the child received after first coming to the CHW, the economic situation of the household, and demographic information about the household. In addition, the data collector verifies which follow-up visit message the caregiver has received from the CHW during the first visit of the child (with a simple fever) to the CHW (the universal, or the conditional follow-up visit message).

Elements of Form 07 – Care-seeking and Demographic Form

Header : The following elements are included in the header of the form : the date when the form is filled out by the data collector, the data collector's ID code, the code of the health center, the case ID for the child, the name of the child, the name of the caregiver.

Sections : This form has 4 sections, each section is explained below. The sections of this form are:

- Section A : The following questions are to be directed to the child's mother/caretaker, and are focused on the child's most recent illness (i.e. the illness for which the child was enrolled 7 days ago).
- Section B : Mother/caretaker/household information.
- Section C : Asset/ownership questions: the following questions are directed towards the mother/caretaker and see information on items owned by the household.

Procedure to fill out Form 07 – Care-seeking and Demographic Form

Header :

1. Fill out the date when Form 07 – Care-seeking and Demographic Form is filled out, the ID code of the data collector, the code of the health center.
2. Fill out the Case ID of the child, the name of the child and the name of the caregiver.

Section A – Care-seeking

The questions in this section are to be directed to the child's mother/caretaker, and are focused on the child's most recent illness. This is the illness for which the child was enrolled in the study 7 days ago. In this section, the data collector asks the caregiver whether they have sought care for the child from someone else, and what were the reasons to seek care elsewhere. In addition, the data collector verifies

which follow-up visit message the caregiver has received from the CHW during the first visit of the child to the CHW (universal or conditional follow-up visit message).

All questions are asked in Swahili to the caregiver of the child that is enrolled in this study. Record the response in the boxes, use one digit per box.

1. Ask : **After your initial visit to the CHW one week ago, did you return with the same child to the CHW ?** If the caregiver says no, record « 0 » in the box and continue to question 2. If the caregiver says yes, record « 1 » in the box and continue to question 1a.
2. If the caregiver responds « yes » to question 1 ask : **After how many days did you return to the CHW?** Record the number of days in the box. If the caregiver does not know the number of days, record « 9 » in the box.
3. Ask : **Why did you return to the CHW ?** Record « 1 » in the box if the caregiver says that the reason for returning to the CHW was that the child's illness was that the child's illness was getting worse, and continue to question 3. Record « 2 » in the box if the caregiver says that the reason for returning to the CHW was that the child was not improving, and continue to question 3. Record « 3 » in the box if the caregiver says that the reason for returning to the CHW was that the CHW directed the caregiver to return, and continue to question 3. Record « 4 » in the box if the caregiver gives another reason for returning to the CHW, and write the reason in the column on the right. Continue to question 3. If there is no information available, record « 9 » in the box.
4. Ask : **Why did you not return to the CHW ?** If the caregiver did not return to the CHW with the child, record the reason why the caregiver did not return here. If the caregiver says that the reason for not returning to the CHW was because the child improved and was no longer ill, record « 1 » in the box. If the caregiver says that the reason for not returning to the CHW was that the caregiver was too busy or had no time, record « 2 » in the box. If the caregiver says that the reason for not returning to the CHW was that (s)he went to a different provider with the child, record « 3 » in the box. If the caregiver gives another reason for not returning to the CHW, record « 4 » in the box and write the reason in the column on the right.
5. Verify which message the CHW gave regarding the follow-up visit of the child to the CHW. Ask: **What did the CHW tell you about a follow up visit with your child ?** Of the caregiver says that, during the first visit of the child to the CHW, the CHW told the caregiver to come back with the child after 2 days (on Day 3), this message is classified as « systematic follow-up visit ». Record « 1 » in the box. If the caregiver says that, during the first visit of the child to the CHW, the CHW told the caregiver to only come back with the child if the condition of the child did not get better, this message is classified as « conditional follow-up visit ». Record« 2 » in the box.
6. Ask : **Before visiting the CHW the first time, did you seek advice or treatment for the child's illness from anyone else ?** If the caregiver has not sought advice or treatment for the child's illness from anyone else before visiting the CHW for the first time, record « 0 » in the box and continue to question 5. If the caregiver has sought advice or treatment for the child's illness from anyone else before visiting the CHW for the first time, record « 1 » in the box and continue to question 4a.
7. Ask : **Where did you see care before visiting the CHW ?** To answer this question, you are asked to record up to three responses in the boxes (one response per box). For the boxes that are not

used (because less than three responses were given by the mother), write “-“, If the caregiver has sought care at a health facility, record « 1 » in the box. If the caregiver has sought care at a hospital, record « 2 » in the box. If the caregiver has sought care at a pharmacy/drug shop, record « 3 » in the box. Record «4 » in the box if the caregiver has sought care at a private clinic/hospital. If the caregiver has sought care at a medicine shop, record « 5 » in the box. Record « 6 » in the box if the caregiver has sought care at a traditional/herbal practitioner. If the caregiver identifies another place where (s)he sought care before visiting the CHW, record « 7 » in the box and specify the place in the column on the right.

8. Ask : **Why did you seek care from somewhere else before visiting the CHW ?** If the caregiver says that the reason for seeking care elsewhere before visiting the CHW was because the sickness of the child was too severe, record « 1 » in the box. If the caregiver says that the reason for seeking care elsewhere before visiting the CHW was because the child had symptoms that are not treated by the CHW (the CHW only treats malaria, diarrhea, and pneumonia), record « 2 » in the box. If the caregiver says that the reason for seeking care elsewhere before visiting the CHW was because of the habit of seeking care from somewhere else, record « 3 » in the box. If the caregiver says that the reason for seeking care elsewhere before visiting the CHW was because the CHW was too far away, record « 4 » in the box. If the caregiver says that the reason for seeking care elsewhere before visiting the CHW was that (s)he does not trust the CHW, record « 5 » in the box. If the caregiver gives another reason for seeking care elsewhere before visiting the CHW, record « 6 » in the box, and write the reason in the column to the right.
9. Ask : **How long did it take for you to get from home to the first place where you sought care?** The objective of this question is to get an indication of the number of minutes that the caregiver and child travelled to the first place where they sought care. Record the response of the caregiver in the two boxes. Use two digits, one digit per box. For example, if the caregiver says (s)he travelled for 15 minutes: record « 15 » in the boxes. If there is no information available, record « 99 » in the boxes.
10. Ask : **What was the total cost of your efforts to seek care before visiting the CHW ?** The objective of this question is to get an indication of the amount of money that the caregiver spent at the first place where the caregiver sought care before visiting the CHW. These costs include the costs of transportation from house to the place where care was sought, any costs related to the consultation/examination/diagnosis, and any costs related to treatment. Use five digits, one digit per box. If the caregiver says 500 FC, record « 00500 ». If the caregiver says 1000 FC, record « 01000 ». If there have not been any costs related for the efforts to seek care before visiting the CHW, record « 00000 ». If there is no information available, record « 99999 » in the boxes.
11. Ask : **After visiting the CHW the first time, did you seek advice or treatment for the child's illness from anyone else ?** If the caregiver has not sought advice or treatment for the child's illness from anyone else after visiting the CHW for the first time, record « 0 » in the box and continue to question 6. If the caregiver has sought advice or treatment for the child's illness from anyone else after visiting the CHW for the first time, record « 1 » in the box and continue to question 5a.
12. Ask : **Where did you see care after visiting the CHW ?** To answer this question, you are asked to

record up to three responses in the boxes (one response per box). For the boxes that are not used (because less than three responses were given by the mother), write "-", If the caregiver has sought care at a health facility, record « 1 » in the box. If the caregiver has sought care at a hospital, record « 2 » in the box. If the caregiver has sought care at a pharmacy/drug shop, record « 3 » in the box. Record «4 » in the box if the caregiver has sought care at a private clinic/hospital. If the caregiver has sought care at a dispensary, record « 5 » in the box. Record « 6 » in the box if the caregiver has sought care at a traditional/herbal practitioner. If the caregiver identifies another place where (s)he sought care after visiting the CHW, record « 7 » in the box and specify the place in the column on the right.

13. Ask : **Why did you seek care from somewhere else after visiting the CHW ?** If the caregiver says that the reason for seeking care elsewhere after visiting the CHW was because the sickness of the child was too severe, record « 1 » in the box. If the caregiver says that the reason for seeking care elsewhere after visiting the CHW was because the child had symptoms that are not treated by the CHW (the CHW only treats malaria, diarrhea, and pneumonia), record « 2 » in the box. If the caregiver says that the reason for seeking care elsewhere after visiting the CHW was because of the habit of seeking care from somewhere else, record « 3 » in the box. If the caregiver says that the reason for seeking care elsewhere after visiting the CHW was because the CHW was too far away, record « 4 » in the box. If the caregiver says that the reason for seeking care elsewhere after visiting the CHW was that (s)he does not trust the CHW, record « 5 » in the box. If the caregiver gives another reason for seeking care elsewhere after visiting the CHW, record « 6 » in the box, and write the reason in the column to the right.
14. Ask : **How long did it take for you to get home from the first place where you sought care?** The objective of this question is to get an indication of the number of hours that the caregiver and child travelled to the first place where they sought care after visiting CHW. Record the response of the caregiver in the two boxes. Use two digits, one digit per box. For example, if the caregiver says (s)he travelled for 15 minutes: record « 15 » in the boxes. If there is no information available, record « 99 » in the boxes.
15. Ask : **What was the total cost of your efforts to seek care after visiting the CHW ?** The objective of this question is to get an indication of the amount of money that the caregiver spent at the first place where the caregiver sought care after visiting the CHW. These costs include the costs of transportation from house to the place where care was sought, any costs related to the consultation/examination/diagnosis, and any costs related to treatment. Use five digits, one digit per box. If the caregiver says 500 FC, record « 00500 ». If the caregiver says 1000 FC, record « 01000 ». If there have not been any costs related for the efforts to seek care before visiting the CHW, record « 00000 ». If there is no information available, record « 99999 » in the boxes.
16. Ask: **At any time while the child was ill, did the child receive any medicine?** If the caregiver says that the child never received any medicine, record « 0 » in the box and continue to question 7. If the caregiver says that the child received medicine, record « 1 » in the box and continue with question 6a.
17. Ask : **Did the child receive medicine for diarrhea?** If the caregiver says the child did not receive medicine for diarrhea, record "0" in the box. If the caregiver says that the child received medicine for diarrhea, record « 1 » in the box. If the caregiver does not know how to respond to

this question (for example, the caregiver says that the child received medicine, but does not know what the medicine was for) record « 8 » in the box. If there is no information available, record « 9 » in the box.

18. Ask : **Did the child receive any antimalarial (medicine for malaria)?** If the caregiver says the child did not receive any malarial, record "0" in the box. If the caregiver says that the child received an antimalarial, record « 1 » in the box. If the caregiver does not know how to respond to this question (for example, the caregiver says that the child received medicine, but does not know what the medicine was for) record « 8 » in the box. If there is no information available, record « 9 » in the box.
19. Ask : **Did the child receive antibiotics (medicine for infection)?** If the caregiver says the child did not receive antibiotics, record "0" in the box. If the caregiver says that the child received antibiotics, record « 1 » in the box. If the caregiver does not know how to respond to this question (for example, the caregiver says that the child received medicine, but does not know what the medicine was for) record « 8 » in the box. If there is no information available, record « 9 » in the box.
20. Ask : **At any time while the child was ill, was the child admitted to a facility (clinic or hospital)?** If the caregiver responds no, record « 0 » in the box and continue to question 8. If the caregiver responds yes, record « 1 » in the box and continue to question 7a.
21. If the caregiver responds that the child was admitted to a facility : Ask : **Which facility ?** If the caregiver says that the child has been admitted to a hospital, record « 0 » in the box. If the caregiver says that the child has been admitted to a health center, record « 1 » in the box. If the caregiver says that the child has been admitted to a dispensary, record « 2 » in the box. If the caregiver says that the child has been admitted to a traditional/herbal practitioner, record « 3 » in the box. If the caregiver says that the child has been admitted to another facility, record « 4 » in the box and write which type of facility in the column on the right.
22. If the caregiver responds that the child was admitted to a facility: Ask : **How long was the child hospitalized ?** Record the number of days that the child was admitted to a facility. Use two digits, one digit per box. For example, if the child has been admitted to a hospital for 6 days, record « 06 » in the boxes. If there is no information available, record « 99 » in the boxes.
23. If the child has died, ask the following questions. If the child has not died, do not ask the following questions and record « 9 » or « 99 » in the boxes.
24. If the child has died: Ask: **Where did the child die?** If the caregiver says that the child died at home, record « 0 » in the box. If the caregiver says that the child died at the hospital, record « 1 » in the box. If the caregiver says that the child died at the health center, record « 2 » in the box. If the caregiver says that the child dies at the traditional/herbal practitioner, record « 3 » in the box. If the caregiver says that the child died elsewhere, record « 4 » in the box and write the place in the column on the right. If there is no information available, record « 9 » in the box.
25. If the child has died : Ask : **When did the child die?** Write the date in the boxes. Use the two boxes on the left to Record the day. Write the month in the two boxes in the middle, and Record the year in the two boxes on the right. Use two digits to Record the day, two digits to Record the month, and two digits to Record the year. For example: write 3 July 2016 as : 03 07 16.
26. If the child has died : Ask : **Family's reported cause of death (write in) ?** Record the response of

the caregiver in the column on the right.

Section B – Mother/Caretaker/Household Information

In this section, information is collected about the demographic characteristics of the mother/caretaker/household. The information that is collected includes the education of the caretaker and husband, religion, ethnicity and income-generating activities that members of the household are engaged in.

1. Ask : **What is your age (in years) ?** Record the age of the caregiver (in years) in the boxes. Use the two boxes, one digit per box. For example, if the caregiver says (s)he is 35 years old, record « 35 » in the boxes. If the caregiver does not know his/her age, help him/her estimating his/her age.
2. Ask: **Can you read?** To evaluate the capacity of the caregiver to read, give the caregiver a sentence and ask him/her to read this out loud. If the caregiver is not able to read the sentence, record « 0 » in the box. If the caregiver is able to read the sentence, but with some difficulty, record « 1 » in the box. If the caregiver is able to read the sentence without any difficulty, record « 2 » in the box.
3. Ask : **Education ?** If the caregiver never has received any education, record « 0 » in the box. If the caregiver has followed a literacy curriculum, record « 1 » in the box. If the caregiver has primary education, record « 2 » in the box. If the caregiver has secondary education, record « 3 » in the box. If the caregiver has university education, record « 4 » in the box. If the caregiver has vocational training, record « 5 » in the box.
4. Ask : **Number of pregnancies ?** Record the number of pregnancies, include any current pregnancy. If the caregiver says she has never been pregnant, record « 0 » in the box. If the caregiver says she has had one or two pregnancies, record « 1 » in the box. If the caregiver says that she has had three or four pregnancies, record « 2 » in the box. If the caregiver says she has had five or six or seven pregnancies, record « 3 » in the box. If the caregiver says that she has had eight or nine or ten pregnancies, record « 4 » in the box. If the caregiver says that she has had more than ten pregnancies, record « 5 » in the box.
5. Ask: **Ethnicity?** If the caregiver says that (s)he identifies as Hemba, record « 0 » in the box. If the caregiver says that (s)he identified as Luba, record « 1 » in the box. If the caregiver says that (s)he identifies as Pygme, record « 2 » in the box. If the caregiver says that (s)he identifies as Tabwa, record « 3 » in the box. If the caregiver says (s)he identifies as Helohelo, record « 4 » in the box. If the caregiver says that (s)he identifies as another ethnicity, record « 5 » in the box and write the ethnicity in the column on the right.
6. Ask : **Religion ?** If the caregiver says (s)he is Christian, record « 0 » in the box. If the caregiver says (s)he is Muslim, record « 1 » in the box. If the caregiver says (s)he is Kinbanguiste, record « 2 » in the box. If the caregiver says (s)he is Kitabala, record « 3 » in the box. If the caregiver says (s)he has another religion, record « 4 » in the box and write which religion in the column on the right.
7. Ask : **Occupation ?** If the caregiver says (s)he does not have an occupation, record « 0 » in the box. If the caregiver says (s)he is a farmer, record « 1 » in the box. If the caregiver says (s)he is a

fisher, record « 2 » in the box. If the caregiver says (s)he works in commerce, record « 3 » in the box. If the caregiver says (s)he is a miner, record « 4 » in the box. If the caregiver says (s)he is a professional, record « 5 » in the box. If the caregiver says that (s)he has another occupation, record « 6 » in the box and write the occupation of the caregiver in the column on the right.

8. Ask : **Marital status ?** If the caregiver says that (s)he is single, record « 0 » in the box and continue to Section C. If the caregiver says (s)he is married, record « 1 » in the box and continue to question 9. If the caregiver says (s)he is divorced, record « 2 » in the box and continue to Section C. If the caregiver says (s)he is widow, record « 3 » in the box and continue to Section C.
9. If the caregiver is married : Ask : **Husband's education ?** If the husband never has received any education, record « 0 » in the box. If the husband has followed a literacy curriculum, record « 1 » in the box. If the husband has primary education, record « 2 » in the box. If the husband has secondary education, record « 3 » in the box. If the husband has university education, record « 4 » in the box. If the husband has vocational training, record « 5 » in the box.
10. If the caregiver is married : Ask : **Husband's occupation ?** If the husband does not have an occupation, record « 0 » in the box. If the husband is a farmer, record « 1 » in the box. If the husband is a fisher, record « 2 » in the box. If the husband works in commerce, record « 3 » in the box. If the husband is a miner, record « 4 » in the box. If the husband is a professional, record « 5 » in the box. If the husband has another occupation, record « 6 » in the box and write the occupation of the husband in the column on the right.

Section C – Asset/Ownership Questions

The following questions are directed toward the mother/caretaker and seek information on items owned by the household, and the characteristics of the house. Make sure to ask all questions to the mother/caretaker. Only observing the house or articles is not sufficient as you could conclude that the household does not own a radio because you don't directly observe one. However, in reality the radio may be stowed away in the cupboard, therefore all questions should be asked.

1. Ask : **What is your household's primary source of drinking water ?** Listen to the response of the caregiver and use the following codes to classify the response. Use two digits to record the code in the boxes, one digit per box. If the caregiver gives a response that can not be classified as any of the responses below, record « 13 » in the boxes and write the response of the caregiver in the column on the right.

01 = Indoor house/piped water
02 = Courtyard/plot piped water
03 = Public/shared piped water
04 = Drill/bored well or pump
05 = Protected dug well
06 = Unprotected dug well
07 = Protected spring water
08 = Unprotected spring water
09 = Collected rainwater
10 = Tanker/delivered water
11 = Small cart/driven tank/barrel
12 = Surface water (river/dam/lake/pond)
13 = Other (Specify _______________)

2. Ask : **What is your household's usual toilet facility ?** Listen to the response of the caregiver and use the following codes to classify the response. Use two digits to record the code in the boxes, one digit per box. If the caregiver gives a response that can not be classified as any of the responses below, record « 10 » in the boxes and write the response of the caregiver in the column on the right.

01 = Flush toilet (sewer)
02 = Flush toilet (septic tank)
03 = Flush toilet (pit/latrine)
04 = Improved self-ventilated pit latrine
05 = Latrine with slab
06 = Basic latrine without slab
07 = Open source/hole/ditch
08 = Composting toilet/latrine
09 = None/outdoors
10 = Other (Specify _______________)

3. Ask : **In this household, are the following items available/present ?** Read each item and record whether the item is available/present in the household.
4. Ask the availability of : **Electricity ?** If not available in the household, record « 0 » in the box. If available in the household, record « 1 » in the box.
5. Ask the availability of : **Radio ?** If not available in the household, record « 0 » in the box. If available in the household, record « 1 » in the box.
6. Ask the availability of : **TV ?** If not available in the household, record « 0 » in the box. If available in the household, record « 1 » in the box.
7. Ask the availability of : **Mobile Phone ?** If not available in the household, record « 0 » in the box. If available in the household, record « 1 » in the box.
8. Ask the availability of : **Generator ?** If not available in the household, record « 0 » in the box. If available in the household, record « 1 » in the box.
9. Ask the availability of : **Chairs ?** If not available in the household, record « 0 » in the box. If available in the household, record « 1 » in the box.
10. Ask the availability of : **Beds ?** If not available in the household, record « 0 » in the box. If available in the household, record « 1 » in the box.
11. Ask the availability of : **Lamps ?** If not available in the household, record « 0 » in the box. If available in the household, record « 1 » in the box.
12. Ask the availability of : **Hoes ?** If not available in the household, record « 0 » in the box. If available in the household, record « 1 » in the box.
13. Ask the availability of : **Sewing machines ?** If not available in the household, record « 0 » in the box. If available in the household, record « 1 » in the box.
14. Ask the availability of : **A Refrigerator ?** If not available in the household, record « 0 » in the box. If available in the household, record « 1 » in the box.
15. Ask the availability of : **Stove/Range ?** If not available in the household, record « 0 » in the box. If available in the household, record « 1 » in the box.
16. Ask the availability of : **A watch ?** If not available in the household, record « 0 » in the box. If

available in the household, record « 1 » in the box.

17. Ask the availability of : **A bike ?** If not available in the household, record « 0 » in the box. If available in the household, record « 1 » in the box.
18. Ask the availability of : **A motorcycle or scooter ?** If not available in the household, record « 0 » in the box. If available in the household, record « 1 » in the box.
19. Ask the availability of : **Bank account ?** If not available in the household, record « 0 » in the box. If available in the household, record « 1 » in the box.
20. Ask : **What is your household's primary source of cooking fuel ?** Listen to the response of the caregiver and use the following codes to classify the response. Use two digits to record the code in the boxes, one digit per box. If the caregiver gives a response that can not be classified as any of the responses below, record « 10 » in the boxes and write the response of the caregiver in the column on the right.

01 = LPG
02 = Natural Gas
03 = Biogas
04 = Kerosene
05 = Coal
06 = Charcoal
07 = Wood
08 = Straw/Thatch
09 = Crop Waste
10 = Other (Specify _______________)

21. Ask : **What is the primary material used for the floor construction ?** Listen to the response of the caregiver and use the following codes to classify the response. Use two digits to record the code in the boxes, one digit per box. If the caregiver gives a response that can not be classified as any of the responses below, record « 10 » in the boxes and write the response of the caregiver in the column on the right.

01 = Earthen floor / sand

02 = Dung
03 = Wood plank/boards
04 = Bamboo
05 = Finished floor boards
06 = Vinyl/Synthetic
07 = Tile
08 = Cement
09 = Carpet
10 = Other (Specify _______________)

22. Ask : **What is the primary material used for the roof construction ?** Listen to the response of the caregiver and use the following codes to classify the response. Use two digits to record the code in the boxes, one digit per box. If the caregiver gives a response that can not be classified as any of the responses below, record « 12 » in the boxes and write the response of the caregiver in the column on the right.

01 = No Roof
02 = Thatch/Palm
03 = Earthen/mud
04 = Bamboo

05 = Wood plank
06 = Cardboard
07 = Plastic Sheet/Tarp
08 = Zinc Sheets
09 = Tiles
10 = Cement
11 = Shingle
12 = Other (Specify _______________)

23. Ask : **What is the primary material used for the exterior wall construction?** Listen to the response of the caregiver and use the following codes to classify the response. Use two digits to record the code in the boxes, one digit per box. If the caregiver gives a response that can not be classified as any of the responses below, record « 13 » in the boxes and write the response of the caregiver in the column on the right.

01 = No Walls
02 = Thatch/Palm/Bamboo
03 = Earthen/mud
04 = Bamboo with mud
05 = Stone with mud
06 = Oven-fired brick
07 = Plywood
08 = Cement
09 = Stone/Mason with mortar
10 = Brick
11 = Cement/block (cinderblock)
12 = Wood shingle/shake
13 = Other (Specify _______________)

Form 08 – Extra Home Visit Form

If the child has a fever, or if the child was treated by the CHW, or if the child was referred for care to a health center during the house visit of the child on Day 7, the data collector and CHW plan a house visit of the child on Day 14. During this house visit, the data collector fills out Form 08 – Extra Home Visit Form. On Form 08 – Extra Home Visit Form we collect information about the health of the child. The data collector takes a several measurements (temperature, MUAC, respiratory rate) that are recorded on Form 08. The CHW conducts an examination of the child to get additional information about the child's health.

Furthermore, if the child has a fever, or if the child was treated by the CHW, or if the child was referred for care to a health center during the house visit of the child on Day 14, the data collector and CHW plan a house visit of the child on Day 28. During this house visit, the data collector fills out a new Form 08 – Extra Home Visit Form. On this new Form 08 – Extra Home Visit Form we collect information about the health of the child. The data collector takes a several measurements (temperature, MUAC, respiratory rate) that are recorded on Form 08. The CHW conducts an examination of the child to get additional information about the child's health.

Elements of Form 08 – Extra Home Visit Form

Header : The following elements are included in the header of the form : the date when the form is filled out by the data collector, the data collector's ID code, the code of the health center, the case ID for the child, the name of the child, the name of the caregiver.

Sections : This form has 4 sections, each section is explained below. The sections of this form are

- Section A : Data collector's measures
- Section B : Results from CHW assessment
- Section C : Classification of the child after assessment
- Section D : Treatment / action taken by CHW

Procedure to fill out Form 08 – Extra Home Visit Form

Header :

1. Fill out the date when Form 08 – Extra Home Visit Form is filled out, the ID code of the data collector, the code of the health center.
2. Fill out the Case ID of the child, the name of the child and the name of the caregiver.
3. **Record number or visit :** record the number of the extra home visit when Form 08 – Extra Home Visit Form is filled out. If this is the house visit of the child on Day 14, record "14" in the boxes, one digit per box. If this is the house visit of the child on Day 28, record "28" in the boxes, one digit per box.
4. **Record child status :** record the vital status of the child in the box. Record « 1 » if the child is alive and present during the house visit on Day 14 or Day 28, and continue filling out Form 08. Record « 2 » if the child died, « 3 » if the child is hospitalized and is never assessed, and « 4 » if the child is lost to follow up (the child is not present). If the child is not present during the planned house visit on Day 14 or Day 28, return the next day to the house of the child to complete Form 06. If « 2 », « 3 », or « 4 » were filled out stop filling out Form 08.

Section A – Data Collector's Measures

In this section, 3 different measures are taken by the data collector. The measures of the child that are taken by the data collector are a supplement to the examination of the child done by the CHW. The CHW conducts his examination of the child as he always does (the examination of the child when he is sick and visits the CHW). In addition to this examination, the data collector measures the child's temperature, MUAC, and respiratory rate. Record the measurements in the boxes, using digits.

4. Use the digital thermometer to measure the body temperature of the child. Record the temperature of the child in the boxes. Write one digit per box and record one decimal.
5. Use the MUAC measurement tool to measure the circumference of the mid-upper arm of the child to examine the nutritional state of the child. Record the circumference of the arm of the child in the boxes. Write one digit per box and record one decimal.
6. Use a stopwatch to record the respiratory rate of the child. Count the number of respiratory movements during one minute. Record the respiratory rate in the boxes. Write one digit per box.

Measuring Child's Body Temperature

1. Turn on the thermometer by pressing on the small round button adjacent to the display. Make sure the display reads "0.0".
2. Clean the thermometer with a piece of alcohol-soaked cotton.
3. Place the thermometer under the armpit of the child and hold it in place until it beeps.
4. After the thermometer beeps, remove it from the armpit of the child and record the temperature in Celsius. When recording on the form, use two digits to the left if the decimal place, and one digit to the right. For example, when the child's temperature is 37.5C, record «37.5» in the boxes.
5. Clean the thermometer with a piece of alcohol-soaked cotton. Only one temperature measurement is required during the house visit.

Measuring Child's Mid-Upper Arm Circumference (using the MUAC tool)

7. Take the left arm of the child.
8. Place the left arm of the child across his chest and ask the caregiver to keep the arm in this position.
9. Use the MUAC tool to determine the midpoint between the olecranon process and the acromion process.
10. Strap the MUAC tool around the arm of the child at the identified midpoint.
11. With the arm of the child hanging down, record the digits that are shown at the arrow in the hole of the MUAC tool (round to the nearest 1 mm).
12. When recording on the form, use two digits to the left of the decimal place, and one digit to the right.

Measuring Child's Respiratory Rate

5. Turn on the stopwatch with the button adjacent to the display. Make sure that the display reads : « 00 :00 ».
6. Start the stopwatch for one minute (60 seconds).

7. Count the respiratory movements of the child during one minute (60 seconds).
8. When recording on the form, use two digits for the respiratory rate of the child.

Section B – Results from the CHW's Assessment

In this section, the data collector records the results from the examination that is done by the CHW. The data collector is present during the examination of the child by the CHW. He follows the examination and records the results in this section. Record the responses in the boxes.

26. Record : **Does the child have fever ?** If the child does not have a fever, write « 0 » in the box and continue to question 2. If the child has a fever, write « 1 » in the box. If there is no information available, write « 9 » in the box. If the child has a fever, write the number of days that the caregiver reported the child having a fever in the box in right column, using one digit.
27. Record : **Does the child have diarrhea ?** If the child does not have diarrhea, write « 0 » in the box and continue to question 3. If the child has diarrhea, write « 1 » in the box. If there is no information available, write « 9 » in the box. If the child has diarrhea, write the number of days that the caregiver reported the child having diarrhea in the box in right column, using one digit.
28. Record : **Does the child have cough ?** If the child does not have cough, write « 0 » in the box and continue to question 4. If the child has cough, write « 1 » in the box. If there is no information available, write « 9 » in the box. If the child has cough, write the number of days that the caregiver reported the child having cough in the box in right column, using one digit.
29. Record : **Does the child have any other complaint** ? If there are no other complaints, write « 0 » in the box and continue to question 5. If there are other complaints, write « 1 » in the box and continue to question 4a. If there is no information available, write « 9 » in the box.
30. Record : **Specify other complaint** ___________. If there are other complaints, record them here. If there are no other complaints, write « RAS » (Rien A Spécifier).
31. Record : **Specify other complaint** ___________. If there are other complaints, record them here. If there are no other complaints, write « RAS » (Rien A Spécifier).
32. Record : **MUAC classification ?** Write the MUAC classification (that is measured by the CHW during the examination of the child) in the box. If the classification is green, write « 1 » in the box. Write « 2 » for yellow, and « 3 » if the MUAC classification is red. If there is no information regarding the MUAC classification, write « 9 » in the box.
33. Record : **Is the child incapable of drinking/eating or breastfeeding?** If no, write « 0 » in the box, write « 1 » in the box if yes. If there is no information available, write « 9 » in the box.
34. Record : **Does the child vomit everything he consumes?** If no, write « 0 » in the box, write « 1 » in the box if yes. If there is no information available, write « 9 » in the box.
35. Record : **Did the child have convulsions or is convulsing now?** If no, write « 0 » in the box, write « 1 » in the box if yes. If there is no information available, write « 9 » in the box.
36. Record : **Is the child unconscious or unresponsive?** If no, write « 0 » in the box, write « 1 » in the box if yes. If there is no information available, write « 9 » in the box.
37. Record : **Is MUAC measurement RED?** If no, write « 0 » in the box, write « 1 » in the box if yes. If there is no information available, write « 9 » in the box.
38. Record : **Is illness more than 14 days, or fever more than 7 days?** If no, write « 0 » in the box,

write « 1 » in the box if yes. If there is no information available, write « 9 » in the box.

39. Record : **Does the child have difficulty breathing while wheezing?** If no, write « 0 » in the box, write « 1 » in the box if yes. If there is no information available, write « 9 » in the box.
40. Record : **Does the child have palmar (or conjunctival) pallor?** If no, write « 0 » in the box, write « 1 » in the box if yes. If there is no information available, write « 9 » in the box.
41. Record : **Is the child often sick?** If no, write « 0 » in the box, write « 1 » in the box if yes. If there is no information available, write « 9 » in the box.
42. Record : **Is the child very weak?** If no, write « 0 » in the box, write « 1 » in the box if yes. If there is no information available, write « 9 » in the box.
43. Record : **Is the child becoming sicker despite care at home?** If no, write « 0 » in the box, write « 1 » in the box if yes. If there is no information available, write « 9 » in the box.
44. Record : **Does the child have visible signs of wasting?** If no, write « 0 » in the box, write « 1 » in the box if yes. If there is no information available, write « 9 » in the box.
45. Record : **Does the child have edema of lower limbs?** If no, write « 0 » in the box, write « 1 » in the box if yes. If there is no information available, write « 9 » in the box.
46. Record : **Does the child have diarrhea with dehydration or blood in stools?** If no, write « 0 » in the box, write « 1 » in the box if yes. If there is no information available, write « 9 » in the box.
47. Record : **Does the child have a fever with generalized skin condition/rash?** If no, write « 0 » in the box, write « 1 » in the box if yes. If there is no information available, write « 9 » in the box.
48. Record : **Record the respiratory rate of the child as measured by the CHW**. Write one digit per box. If the respiratory rate is not recorded, write « 00 » in the boxes.
49. Record : **Does the child have a fever (hot to touch or fever within two days)?** If no, write « 0 » in the box and continue to Section C. Write « 1 » in the box if yes and continue to question 5a. If there is no information available, write « 9 » in the box.
50. If the child has a fever: Record : **What is the RDT result ?** If there is no result recorded of a RDT (RDT is not done, box is blank), write « 0 » in the box. If the result of the RDT is positive, write « 1 » in the box. If the result of the RDT is negative, write « 2 » in the box.

Section C – Classification of the Child After Assessment

In this section the classification of the child after the examination by the CHW is recorded. The CHW determines the classification of the child based on his examination. The data collector records information about the classification on this form. This section seeks to collect information about the following classifications: malaria, diarrhea, pneumonia, moderate malnutrition, fever, and cough. Record the responses in the boxes, using one digit.

9. Record the classification of the child after examination by the CHW of **malaria**. If the child does not have malaria, record « 0 » in the box and continue to question 2. If the child has malaria, record « 1 » in the box and continue to question 1a. If there is no information available, record « 9 » in the box.
10. If the CHW determines that the child has malaria, record whether the child is a **confirmed or a presumed case of malaria**. Record « 1 » if it is a confirmed case (result of the TDR is positive). Record « 2 » if it is a presumed case (result of the RDT is not available).

11. Record the classification of the child after examination by the CHW of **diarrhea**. If the child does not have diarrhea, record « 0 » in the box. If the child has diarrhea, record « 1 » in the box. If there is no information available, record « 9 » in the box.
12. Record the classification of the child after examination by the CHW of **pneumonia**. If the child does not have pneumonia, record « 0 » in the box. If the child has pneumonia, record « 1 » in the box. If there is no information available, record « 9 » in the box.
13. Record the classification of the child after examination by the CHW of **moderate malnutrition**. If the child does not have moderate malnutrition, record « 0 » in the box. If the child has moderate malnutrition, record « 1 » in the box. If there is no information available, record « 9 » in the box.
14. Record other classifications of the child after examination by the CHW using **other (describe)**. Write other classifications on the form. If there are no other classifications after examination by the CHW, write « RAS » (Rien A Spécifier).
15. Record the classification of the child after examination by the CHW of **fever (with or without cough)**. If the child does not have fever (with or without cough), record « 0 » in the box. If the child has fever (with or without cough), record « 1 » in the box. If there is no information available, record « 9 » in the box.
16. Record the classification of the child after examination by the CHW of **cough**. If the child does not have cough, record « 0 » in the box. If the child has cough, record « 1 » in the box. If there is no information available, record « 9 » in the box.

<u>Section D – Treatment/action taken by CHW</u>

In this section information is recorded about the treatment of the child after examination by the CHW. The CHW determines the treatment of the child based on his examination of the child. The data collector records the information on this form. Record the responses in the boxes.

10. Record: **Antimalarials given?** Record whether the CHW has given any antimalarials to the child. If the CHW did not give any antimalarials to the child, record « 0 » and continue to question 2. Record « 1 » if the CHW did give any antimalarials to the child, and continue to question 1a. If there is no information available, record « 9 » and continue to question 2.
11. If the CHW gave any antimalarials, record : **Describe______________.** Describe which type of antimalarials the CHW gave to the child. Record « RAS » (Rien A Spécifier) if the CHW gave antimalarials to the child, but there is no information available about which type of antimalarials.
12. Record: **Antibiotics given?** Record whether the CHW has given any antibiotics to the child. If the CHW did not give any antibiotics to the child, record « 0 » and continue to question 3. Record « 1 » if the CHW did give any antibiotics to the child, and continue to question 2a. If there is no information available, record « 9 » and continue to question 3.
13. If the CHW gave any antibiotics, record : **Describe______________.** Describe which type of antibiotics the CHW gave to the child. Record « RAS » (Rien A Spécifier) if the CHW gave antibiotics to the child, but there is no information available about which type of antibiotics.
14. Record: **ORS/Zinc given?** Record whether the CHW has given any ORS/Zinc to the child. If the

CHW did not give any ORS/Zinc to the child, record « 0 » and continue to question 4. Record « 1 » if the CHW did give any ORS/Zinc to the child, and continue to question 3a. If there is no information available, record « 9 » and continue to question 4.

15. If the CHW gave any ORS/Zinc, record : **Describe_______________.** Describe which type of ORS/Zinc the CHW gave to the child. Record « RAS » (Rien A Spécifier) if the CHW gave ORS/Zinc to the child, but there is no information available about which type of ORS/Zinc.
16. Record : **Paracetamol given ?** Record whether the CHW has given any paracetamol to the child. If the CHW did not give any paracetamol to the child, record « 0 ». Record « 1 » if the CHW gave paracetamol to the child. If there is no information available, record « 9 » in the box.
17. If you fill out Form 08 – Extra Home Visit Form during the house visit of the child on Day 14: Determine: Did the child have fever, or was the child treated, or was the child referred for care to a health center? If no (the child did not have a fever, the child was not treated by the CHW, and the child was not referred for care to a health center), record « 0 » in the box and contact the child's caregiver on Day 30 to record the vital status of the child. If yes to at least one criteria (the child has a fever, or the child is treated by the CHW, or the child was referred for care to a health center), record « 1 » in the box and plan the house visit of the child on Day 28.
    If you fill out Form 08 – Extra Home Visit Form during the house visit of the child on Day 28:
18. Determine: **Did the child have fever, or was the child treated, or was the child referred for care to a health center?** If no (the child did not have a fever, the child was not treated by the CHW, and the child was not referred for care to a health center), record « 0 » in the box and contact the child's caregiver on Day 30 to record the vital status of the child. If yes to at least one criteria (the child has a fever, or the child is treated by the CHW, or the child was referred for care to a health center), record « 1 » in the box and contact the child's caregiver on Day 30 to record the vital status of the child.
19. If C6 = 0 (the child does not have fever during the Day 14 or Day 28 visit), ask the following question: **How many days ago did your child last have fever?** Record the answer to this question in number of days in the boxes using two digits. Record « 00 » if the child last had fever today, « 01 » to « 28 » for the numbers of days and « 99 » if the mother or caregiver does not know how many days ago the child last had fever.

Form changes

Form 03 : Initial Visit Extraction Form

1. On July 1, 2016, all data collectors were instructed to record the respiratory rate of the child as was recorded on the iCCM Child Form during the first visit of the child to the CHW. The CHW counts the respiratory rate if a child presents at the CHW site with « cough » as complaint. The following question was added to Form 03 – Initial Visit Extraction Form: **Record the respiratory rate.** The data collectors were instructed to record "00" if the child's respiratory rate was not recorded on the iCCM Child Form.

Form 06 : Day 7 Assessment Form

1. In the week of November 30, 2015, all data collectors were instructed to ask an additional question if the child did not have a fever during the Day 7 visit. The question was added to the end of the form, and is only asked when C6 = 0 (the child does not have fever during the Day 7 visit). If that is the case, the data collector asks the mother or caregiver: "**How many days ago did your child last have fever?".** The answer to this question is noted in number of days using two digits. This decision was taken following the conclusion that some of the Day 7 visits were actually conducted on later dates due to the absence of the CHW or the mother or caregiver on Day 7.

Form 07 : Care-seeking and Demographic Form

1. On November 6, 2015, all data collectors were instructed to note the answers to questions 4c and 5c in minutes, instead of hours. This decision was taken during the first meeting with all the data collectors when it became clear that mothers travelled for minutes, and not for hours to seek care for their sick child (before, or after visiting the CHW).
2. On March 1, 2016, all data collectors were instructed to ask an additional question to the caregivers if the child had received any medication during this illness. If the caregiver responds « yes » (Question 6=1), the data collectors were instructed to ask question 6a (did the child receive medication against diarrhea), question 6b (did the child receive medication against malaria), and question 6c (did the child receive antibiotics). Now the following question 6d was added: « **did the child receive paracetamol?** ». The decision to add this question to Form 07 was made following a discussion between JHSPH's PI, representatives of the CDC and the PI from the TRAction study in Ethiopia.
3. On March 1, 2016, all data collectors were instructed to ask an additional question to the caregivers if the child had received paracetamol during this illness (question 6d=1). The data collectors were instructed to ask the following question: "**did the child take paracetamol during the past 8 hours?**" The decision to add this question to Form 07 was made following a discussion between JHSPH's PI, representatives of the CDC and the PI from the TRAction study in Ethiopia.

Form 08 : Extra Visit Form

1. In the week of November 30, 2015, all data collectors were instructed to ask an additional question if the child did not have a fever during the Day 14 or the Day 28 visit. The question was added to the end of the form, and is only asked when C6 = 0 (the child does not have fever

during the Day 14 or Day 28 visit). If that is the case, the data collector asks the mother or caregiver: “**How many days ago did your child last have fever?”.** The answer to this question is noted in number of days using two digits. This decision was taken following the conclusion that some of the Day 14 and Day 28 visits were actually conducted on later dates due to the absence of the CHW or the mother or caregiver on Day 14 or Day 28.

End of Enrollment and Follow Up Decisions

CHWs

- CHWs will continue to read recruitment script to the caregivers of eligible children through the end of day November 28, 2016
- CHWs will continue to record the result of those recruitment efforts on Form 01, through the end of the day November 28, 2016

Data Collectors

- Data collectors will conduct enrollment visits through the end of the day December 5, 2016
- Data collectors will also complete any previously scheduled Day 7 visits, Day 14 visits, Day 28 visits, or Day 30 Status Check visits that have a schedule date on or before December 5, 2016
- When scheduling Day 30 status check dates on Form 02, the Data collector should record 05/12/16 if the estimated/calculated day falls on or after 05/12/16
- On December 5, 2016, they need to fill out all remaining Day 30 status boxes (last box on Form 02), by confirming the status of all children remaining (through visit to the home of the child, or by asking the CHW to confirm the vital status of the child).
- On December 5, 2016, the Data Collectors should retrieve all Form 01 from the CHWs.
